# Electromagnetic metamaterial agent

**DOI:** 10.1038/s41377-024-01678-w

**Published:** 2025-01-01

**Authors:** Shengguo Hu, Mingyi Li, Jiawen Xu, Hongrui Zhang, Shanghang Zhang, Tie Jun Cui, Philipp del Hougne, Lianlin Li

**Affiliations:** 1 State Key Laboratory of Advanced Optical Communication Systems and Networks, School of Electronics, Peking University, Beijing, 100871 China; 2 National Key Laboratory for Multimedia Information Processing, School of Computer Science, Peking University, Beijing, 100871 China; 3 State Key Laboratory of Millimeter Waves, Southeast University, Nanjing, 210096 China; 4 Pazhou Laboratory (Huangpu), Guangzhou, Guangdong 510555 China; 5 Univ Rennes, CNRS, IETR - UMR 6164, F-35000 Rennes, France

**Keywords:** Photonic devices, Imaging and sensing

## Abstract

Metamaterials have revolutionized wave control; in the last two decades, they evolved from passive devices via programmable devices to sensor-endowed self-adaptive devices realizing a user-specified functionality. Although deep-learning techniques play an increasingly important role in metamaterial inverse design, measurement post-processing and end-to-end optimization, their role is ultimately still limited to approximating specific mathematical relations; the metamaterial is still limited to serving as proxy of a human operator, realizing a predefined functionality. Here, we propose and experimentally prototype a paradigm shift toward a metamaterial agent (coined metaAgent) endowed with reasoning and cognitive capabilities enabling the autonomous planning and successful execution of diverse long-horizon tasks, including electromagnetic (EM) field manipulations and interactions with robots and humans. Leveraging recently released foundation models, metaAgent reasons in high-level natural language, acting upon diverse prompts from an evolving complex environment. Specifically, metaAgent’s cerebrum performs high-level task planning in natural language via a multi-agent discussion mechanism, where agents are domain experts in sensing, planning, grounding, and coding. In response to live environmental feedback within a real-world setting emulating an ambient-assisted living context (including human requests in natural language), our metaAgent prototype self-organizes a hierarchy of EM manipulation tasks in conjunction with commanding a robot. metaAgent masters foundational EM manipulation skills related to wireless communications and sensing, and it memorizes and learns from past experience based on human feedback.

## Abstract (web summary)

metaAgent has the capability of reasoning and cognition to autonomously plan and execute complex electromagnetic manipulation tasks.

## Graphical abstract

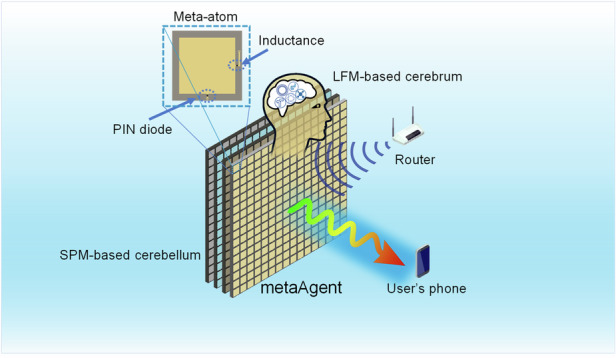

## Introduction

Since the turn of the millennium^1–3^, metamaterials have revolutionized the manipulation of waves across scales and wave phenomena. Starting with passive devices aimed at exotic wave manipulations like anomalous refraction^1–5^, invisibility cloaking^6–8^ or perfect lensing^9,10^, the field evolved toward programmable devices^11^, in particular programmable metasurfaces^12,13^, aimed at providing increasingly applied functionalities in areas like wireless communications and sensing. Latest generations of programmable metasurfaces are endowed with sensors so that they can self-adaptively realize a user-defined functionality^14–19^. Currently, a plethora of emerging deep-learning techniques drives new developments in the research on metamaterials, ranging from their inverse design^20–22^ via the post-processing of measured data in sensing applications to end-to-end optimized metamaterial devices^23–26^. However, the role of deep-learning in the field of metamaterials is to date limited to approximating specific mathematical functions that are not analytically tractable^27,28^. At the same time, the metamaterial devices remain to date proxies of human operators, niched to realizing a predefined functionality, possibly with the help of the aforementioned deep-learning tools. The functionality must be chosen by the human operator and prepared in advance (e.g., via inverse design or training of an artificial neural network); during runtime, the functionality cannot swiftly be modified. Most glaringly, existing metamaterial devices lack the reasoning and cognitive capabilities with which their human operators are endowed to understand environmental cues and accordingly plan their actions themselves. Ideally, metamaterials would be autonomous reasoning agents that monitor their surroundings via diverse sensing modalities, understand the evolution of their complex surrounding, self-organize their actions accordingly across long horizons, and execute their planned actions (possibly in collaboration with robotic entities). Advanced wave manipulation skills related to sensing and wireless communications based on programmable metasurfaces are clearly a prerequisite for such metamaterial agents’ sensing modalities as well as the execution of their planned actions and communications with robotic entities.

Humans reason in natural language which provides a powerful high-level abstraction, humans have a broad set of expertise in diverse domains, and humans are single-shot or few-shot learners. Recent releases of large-capacity foundation models (LFMs)^29^, in particular large language models (LLMs) like the GPTs^30–32^, BERT^33^, Gemma^34^, LLaMA^35^ and Mistral^36^, broadly attracted public attention because of their unprecedented abilities to reason in natural language, to adapt their broad knowledge learned from internet-scale data to a wide variety of downstream tasks, and to learn unseen knowledge with a single or a few shots^37^. The availability of pre-trained LFMs makes it possible for downstream users to fine-tune an LFM for a desired task with very little task-specific data, or even to merely provide a few context-clarifying prompts in natural language. LFMs have successfully been applied to areas including task planning^38,39^, computer code generation^40^, protein design^41^, and information extraction from materials science literature^42^. Within the fields of electromagnetism and metamaterials, first studies using LFMs already demonstrated the semantic regularization of inverse problems^43^, the fine-tuning of LFMs to inverse-design metasurfaces^44^, and the configuration of a programmable metasurface according to a suitable prompt in natural language^45^. Some of these studies provide early indications that the abstraction to natural language provides superior generalization and noise-robustness capabilities compared to more conventional deep-learning tools^43,44^. Nonetheless, ultimately these early studies replace artificial neural networks or other algorithms implementing a specific functionality by LFMs; the LFMs in refs. ^43–45^ are hence not used for reasoning in natural language and the metamaterials in refs. ^43–45^ are not autonomous metamaterial agents. Yet, in sight of the capabilities and availability of pre-trained LFMs, the latter appear ideally suited to realize the aforementioned vision of a metamaterial agent reasoning in natural language.

Here, we propose and experimentally prototype such a reasoning metamaterial agent which we coin metaAgent. Our experiments take place in a real-life indoor environment and emulate an assisted-living context, where metaAgent assimilates environmental cues via various sensors (e.g., audio, text, visual, radio) and then independently orchestrates its actions accordingly. For instance, upon receiving audio input revealing that a human is feeling unwell, metaAgent may decide to cascade a variety of EM manipulation tasks using its programmable metasurfaces to proactively seek out supplementary focused observations. Specifically, this could involve wirelessly localizing the human and then performing fine-grained monitoring of the human’s respiration and heartbeat. Based on the obtained results, metaAgent may decide that a robotic entity should be dispatched to deliver drugs to the human. metaAgent would then use its programmable metasurfaces to establish a strong wireless communication channel with the robotic entity to transmit those instructions. As the robot moves, metaAgent would constantly wirelessly localize the robot using a programmable metasurface and adapt the configuration of a programmable metasurface to maintain a strong wireless link with the robot. Importantly, this highly complex multi-task coordination across long horizons is achieved by metaAgent without any human intervention. However, metaAgent can learn to improve based on human feedback in natural language.

**Fig. 1:**
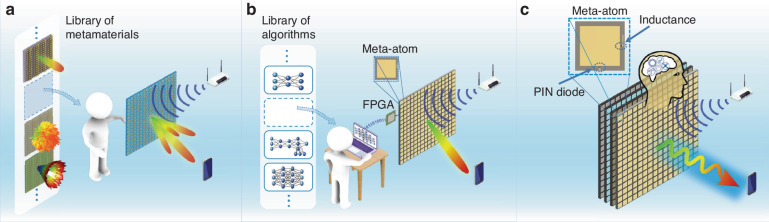
Conceptual illustrations of a passive metasurface, a computation-enabled active metasurface, and our metaAgent. **a** Passive metasurface: one needs to manually change the metamaterial itself, respectively, when the operational environment or demanded task is changed. **b** Computation-enabled active metasurface: one needs to manually change the governing algorithms (which could be artificial neural networks). **c** Our metaAgent: in contrast, the metaAgent is capable of autonomously planning and executing EM wave manipulations in response to an evolving environment and human feedback because it is endowed with the ability to reason in natural language. Specifically, the metaAgent’s cerebrum features a multi-agent discussion in natural language leveraging state-of-the-art LFMs in order to understand environmental cues, plan suitable actions and execute them based on EM wave manipulations with programmable metasurfaces. In addition, details about the meta-atom have provided in Methods and Supplementary Note 2

On the hardware level, metaAgent’s EM wave manipulation capabilities leverage programmable metasurfaces^12^ which are by now established hardware. The key contribution of the present work is that the metasurfaces are no longer limited to static responses or predefined choices of functionalities. Instead, as highlighted in Fig. 1, we endow meta-agent with the capability of reasoning in natural language to enable autonomous multi-modal multi-task operations which pivotally rely on EM wave manipulations with programmable metasurfaces. To reason in natural language, metaAgent’s cerebrum features a multi-agent discussion mechanism in natural language. Each agent has a specific expertise (e.g., planning, coding, etc.) and is based on an LFM that has received a context demonstration, i.e., it has been briefed in natural language about its role. Importantly, we are hence not performing gradient updates or fine-tuning of the LFMs to conceive our LFM-based expert agents. This remarkably frugal approach is enabled by the public availability of LFMs and their few shot learning capabilities^37^. Moreover, the high-level abstraction of the multi-agent discussion in natural language may provide pivotal advantages in terms of generalization and robustness, as hinted at earlier. metaAgent’s cerebrum manages perceptual, planning and execution steps in a hierarchical manner, mirroring the sequential focus of human attention^46^.

## Results

### Operational principle of metaAgent

**Fig. 2:**
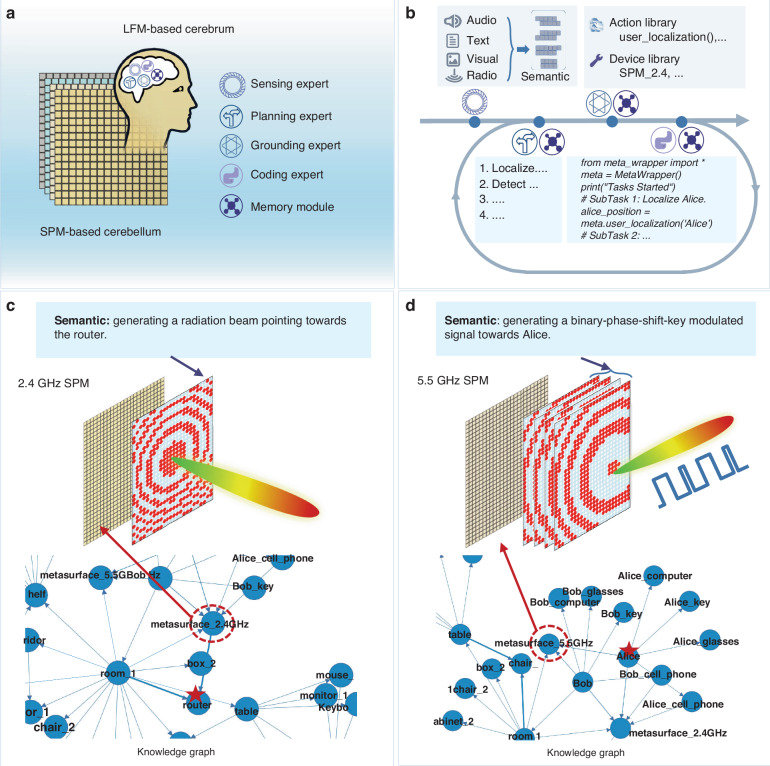
System configuration and operational principle of metaAgent. **a** System configuration: the metaAgent takes a collection of SPMs as its cerebellum, while its cerebrum is composed of a multi-agent discussion between four different domain experts (sensory expert, planning expert, grounding expert and coding expert). Besides, a memory module is introduced into the cerebrum for saving the knowledge graph, 3D visual-semantic map and past experiences. See Fig. S1 for further details. **b** Operational principle: metaAgent autonomously accomplishes the user’s command by taking sequentially the four-step operations: (i) the sensory expert summons the multi-modality sensor data (radio, audio, text, image) and synthesizes the information in natura language, (ii) the planning expert generates a body of executable subtasks, (iii) the grounding expert assigns each subtask with an action function and associated devices, (iv) the coding expert generates the action policy. The coding expert produces two types of outputs in natural language: one ‘external’ output for the communication with the external human user, and one ‘inner’ output for consideration by the planning and grounding experts. See Fig. S3 for further details. **c**, **d** are two experimental examples of semantic coding patterns. Here, given the knowledge graph, the semantic coding patterns convey the semantics of the SPM with a given space or space-time coding pattern is responsible for ‘generating a radiation beam pointing towards the router’ and ‘generating a binary-phase-shift-key modulated signal for Alice’, respectively. Additionally, the SPM’s deployment in our lab has been detailed in Fig. S2

Our metaAgent is an entity that autonomously engages with an evolving uncertain environment via intricate interactions between EM waves, matter and information. As sketched in Fig. 2a, metaAgent is composed of two constituting parts: one is the cerebellum for executing specific EM wave manipulation tasks using semantically programmable metasurfaces (SPMs), and the other one is the LFM-based cerebrum for delegating a hierarchy of executable subtasks to the cerebellum (see Supplementary Note 1). The hardware of SPMs resembles that of conventional programmable metasurfaces^12^ (see Methods and Supplementary Note 2), but we specifically use the terminology SPM here to highlight that the control coding patterns (which we refer to as semantic coding patterns) embody prompts formulated in high-level natural language as a result of the reasoning process in metaAgent’s cerebrum, rather than a low-level sequence of binary digits as in conventional programmable metasurfaces^12^.

On the basis of multi-modal inputs (text, voice, image, microwave signals), metaAgent’s cerebrum launches an action plan that involves commanding the SPM for various purposes, including the acquisition of supplementary sensory data, the commanding of a robotic entity, etc. This requires an abstract understanding of environmental cues such as spoken instructions, long-horizon reasoning over the required order of various actions, and knowledge of both the environment and metaAgent’s capabilities. With respect to the SPM, the cerebellum can be characterized by a function $\pi$,

1 $$\left({{ID}}_{{\rm{meta}}},{\boldsymbol{C}}\right)=\pi (l,{other\; inputs;KG})$$

where $\pi$ converts natural-language prompts $l$ and other inputs based on stored knowledge (in the form of a knowledge graph (KG) here) into an index ${{ID}}_{{\rm{meta}}}$ and space (or space-time) coding pattern ${\boldsymbol{C}}$ of the chosen SPM. Here, the policy function $\pi$ is constructed as a pipeline, where the natural-language prompts $l$ and other inputs from *KG* are first inputted to LLMs, and the LLMs generates executable Python code after series of reasoning. Then, in this pipeline the code generated by LLMs is executed to control the coding pattern ${\boldsymbol{C}}$ of the target SPM via the Field Programmable Gate Array (FPGA). Of course, the major challenge lies in representing $\pi$ due to the cross-modality nature of inputs and knowledge, and the uncertain surrounding environment. metaAgent’s cerebrum tackles this challenge by a “chain-of-thought” that is implemented via discussions in natural language^47^ of the following four domain experts (see Methods):

1. **Sensing expert**. The sensing expert continuously takes multi-modal data as input and outputs the semantic result for the planning expert.
2. **Planning expert**. The planning expert, drawing on knowledge provided in the memory module, analyzes the natural-language input from the sensing expert or the coding expert, and decomposes it into a series of feasible subtasks.
3. **Grounding expert**. The grounding expert receives the planning expert’s list of subtasks and assigns suitable action functions and associated devices for each subtask.
4. **Coding expert**. The coding expert takes as input the triplet of intended goal, required action function and devices in natural language, then writes python code that runs on the host computer using the known functions.

Whenever an expert’s output causes an error at the subsequent stage, the former expert is asked to produce a new output, until the output no longer produces a subsequent error. For instance, if the python code generated by the coding expert produces an error, metaAgent re-calls the coding expert who will generate an improved code. As mentioned, the memory module is an important part of metaAgent’s cerebrum, accessed by the planning expert, the grounding expert and the coding expert. The memory module contains an action library and a memory library (see Methods).

Before considering increasingly complex environmental cues and situations in the subsequent sections, we illustrate the semantic coding patterns for two relatively simple examples. In Fig. 2c, the natural-language prompt “generating a radiation beam pointing toward a router” is converted into a spatial coding pattern of the 2.4 GHz programmable metasurface using the KG which contains information about the location of the router and the programmable metasurface. The spatial coding pattern is inverse-designed by the metaAgent using the modified Gerchberg-Saxton (G-S) algorithm^48^ (see Supplementary Note 2 for details). In Fig. 2d, the natural-language prompt “generating a binary-phase-shift-key modulated signal towards Alice” is converted into a space-time coding pattern of the 5.5 GHz programmable metasurface. Here, while the location of the 5.5 GHz programmable metasurface is looked up by the metaAgent in the KG, the location of Alice and Bob must be determined first, and subsequently a suitable space-time coding pattern of the programmable metasurface is inverse-designed by the metaAgent using again the modified G-S algorithm.

We have examined the performance of the planning and grounding experts over open-vocabulary natural-language commands in a comprehensive series of experiments in our real-life highly complex laboratory indoor environment featuring bookcases, desks, chairs, computers, electronic instruments, wireless routers, and so on. In our experiments, we consider 100 language commands with different levels of complexity (see details in Supplementary Note 7), and each command is implemented 100 times in different settings of our environments. Experimental results are reported in Table 1, revealing that metaAgent can understand the user’s intention and formulate the correct policy, and that the grounding expert can obtain a success rate exceeding 97% on average. These results indicate that our metaAgent is capable of digesting diverse spoken instructions, converting them into suitable feasible actions involving the available programmable metasurfaces.

**Table 1:** Success rates of our metaAgent over 100 language commands with different complexities

Instructions		Steps	Success Rate (%)			
			TP	AS&DA	CG	TE
**simple instructions**	Where is the table?	1	100	100	100	100
	Where is Alice?	1	100	100	100	98
	Where is my cell phone?	1	97	100	98	93
	Where is robot A?	1	98	100	98	94
	Please locate the router.	1	96	100	98	93
	Please check Bob’s breathing.	2	98	100	94	90
	What happened to Alice?	2	94	100	96	89
	Please check Bob’s health status.	2	90	98	92	84
	Enhance the Wi-Fi signal in the corridor.	3	97	100	98	90
	My cell phone cannot get a signal, please help me.	3	98	99	100	88
	…	…	…	…	…	…
	**Average performances of all 50 simple commands**		**97.4**	**99.2**	**96.9**	**90.2**
**complex instructions**	Please check the respiration rate of Bob and Alice.	4	94	98	99	87
	What are Bob and Alice doing?	4	96	99	98	92
	Robot A, please see me.	5	88	98	96	78
	Robot A, take me to the corridor.	7	85	96	90	72
	Please send this picture to Alice’s computer.	4	89	99	95	86
	Let the robot A go to the pillbox and bring me my antihypertensive pills.	8	85	97	91	73
	Please bring Bob his antihypertensive pills.	8	78	95	92	71
	Let robot A to take the wooden block Bob’s holding to Alice.	9	75	95	89	72
	Let robots A and B go to room A and the corridor respectively.	10	85	96	93	78
	Bob needs help, his pressure is really high.	9	72	96	85	67
	…	…	…	…	…	…
	**Average performances of all 50 complex commands**		**84.2**	**97.8**	**91.6**	**72.3**

*Steps* the number of steps required to complete the instruction, *TP* task planning, *AS&DA* action selection and device allocation, *CG* code generation, *TE* task execution

In addition, we also report the average performances of all involved 50 simple and 50 complex commands, respectively. Note that only 10 simple and 10 complex commands are listed here due to the limited space

### Experimental results of autonomous EM manipulations

**Fig. 3:**
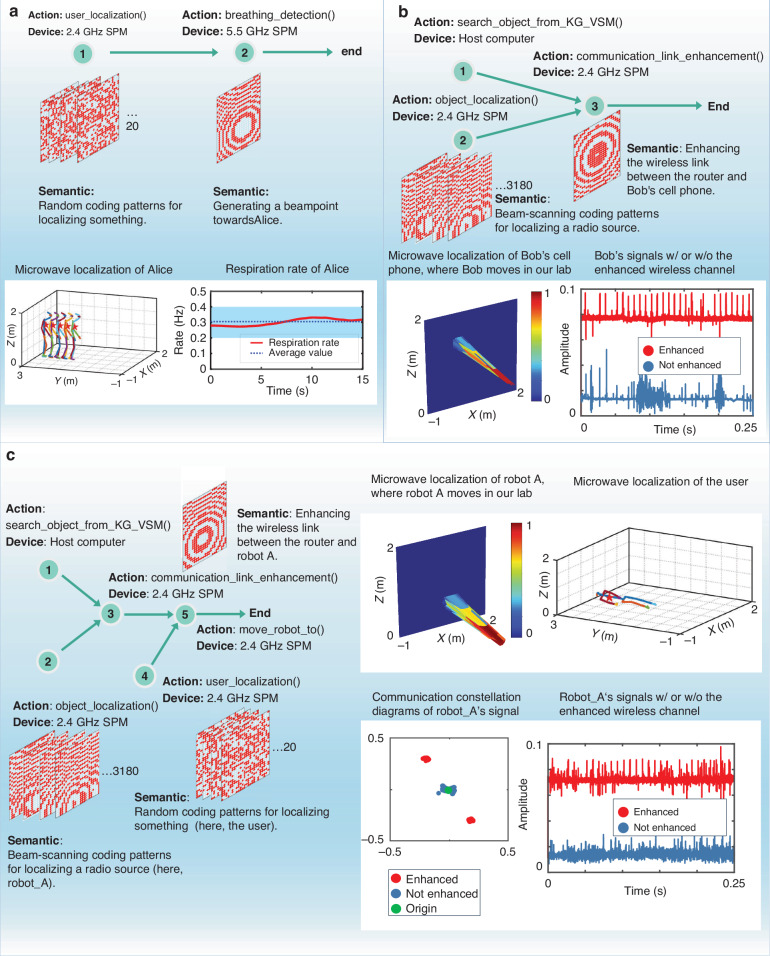
Three selected experimental results of autonomous EM manipulation, where the demanded tasks from a user’s voice commands. **a** ‘please check Alice’s breathing status’ : (Top) the graph representation of planned subtasks, where two subtasks are organized in a sequential manner. For each subtask, the action function, SPM device, and the required semantic coding pattern are marked. For subtask-1, 2.4 GHz SPM and 20 random coding patterns are assigned for reconstructing the 3D human skeleton through the microwave imaging strategy; for subtask-2, 5.5 GHz SPM and a focusing coding pattern are assigned for remotely detecting the subject’s breathing rate via the microwave sensing technique. (bottom) the executed results corresponding to planned two subtasks, i.e., the Alice’s location has been marked with red stars in the reconstructed human skeletons for subtask-1, while a 15 s-length respiration rate is plotted for subtask-2. **b** ‘my cell phone cannot get a signal, please help me’ : (Top) the graph representation of planned three subtasks. For subtask-1, the router can be easily localized by looking up the knowledge map or visual-semantic map, and thus there is no need to use the SPM; for subtask-2, the user’s cell phone is localized via the beam-scanning strategy, where 3180 focusing coding patterns are involved; for subtask-3, the wireless link between the router and the user’s cell phone is established and enhanced by using a 2.4 GHz SPM. (bottom) the executed results corresponding to planned three subtasks: the microwave localization of the user’s cell phone for subtask-2, and a 0.25 s-length wireless signals at the subject for subtask-3. One can see that the wireless signal of the user has been remarkably enhanced. **c** ‘robot A, please see me’ : (left) the graph representation of planned five subtasks. For subtask-3 and 5, they share the same semantic coding pattern for the enhanced wireless communication between the router and robot_A. Besides, for subtask-5, it involves the information transfer from metaAgent to robot_A, which the transferred information is about the user’s localization. (right) the executed results corresponding to four subtasks: microwave localization of robot_A for subtask-2, microwave reconstruction of user’s 3D skeleton for subtask-3, a 0.25 s-length wireless signals at the subject for subtask-3, and the constellation of wireless communication between robot_A and router

We now provide three representative experimental results in Fig. 3 to demonstrate the metaAgent’s capabilities in accomplishing the user’s intention through the autonomous EM manipulation in real-world settings. In this section, the user communicates their intention in natural language; more complex scenarios are considered in the subsequent section.

First, we consider a relatively simple case to illustrate the operational pipeline of metaAgent, where a user somewhere in our laboratory initiates a voice request, i.e., ‘please check Alice’s breathing status’. In light of Fig. 2b, the metaAgent, after receiving the command through the in-built microphone, follows a three-step operation pipeline: (i) plan a body of subtasks through the planning expert, (ii) assign each subtask with an action function and associated devices through the grounding expert, (iii) implement sequentially the planned subtasks with the coding expert. For the first step, the metaAgent produces a task decomposition in natural language: (1) localize Alice, (2) detect Alice’s breathing rate, (3) end. It is clear that the original ambiguous instruction has been broken up into two sequential subtasks that match well the metaAgent’s capabilities (see Fig. 3a). Afterwards, through consultation with the grounding expert, the metaAgent assigns to each subtask an action function and associated devices from FunctionLibrary and DeviceLibrary, respectively. For subtask-1, the chosen action function and device are ‘user_localization’ and the 2.4 GHz SPM, respectively. Similarly, ‘breathing_detection’ and the 5.5 GHz SPM are chosen for subtask-2. Note that the metaAgent automatically chooses a set of 20 random coding patterns for the action function ‘user_localization’ while it chooses the focusing coding pattern for ‘breathing_detection’ in order to suppress unwanted clutter by boosting the signal-to-noise ratio. These two actions are sequentially implemented and corresponding results are reported in the bottom of Fig. 3a, indicating that Alice is healthy in terms of her respiration rate, i.e., 0.3 Hz. These results demonstrate that the metaAgent can autonomously perform the intended EM manipulation task without human intervention.

Next, we consider two more challenging cases for which the voice instruction from the user is ‘my cell phone cannot get a signal, please help me’ and ‘robot A, please see me’, where robot A is a mobile robot in our laboratory environment. These two cases are more challenging than the first case since the spoken user instruction is more ambiguous, and the subtasks planned by the metaAgent are arranged in a more complicated tree structure rather than a simple sequential manner. As shown in Fig. 3c, the metaAgent responds with 5 subtasks: (1) find the router, (2) localize robot A, (3) establish the wireless link between robot A and router, (4) localize the user, (5) move robot A to the user, (6) end. Similar to above, the metaAgent needs to drive a SPM for implementing a certain kind of EM manipulation for some subtasks. For instance, for subtask-4, the 2.4 GHz SPM is utilized while robot A moves towards the user. Note that the router has been saved in the memory module (i.e., KG or VSM) so there is no need for the metaAgent to localize the router using a microwave sensing technique. More details have been provided in Fig. 3b, c. The results allow us to conclude that the metaAgent can understand user’s open-vocabulary requests, self-organize in order the required subtasks, and autonomously accomplish a diversity of human-robot interactions in real-world settings.

Before closing this section, we examine the metaAgent’s performance in terms of the success rate which characterizes the fraction of times that the metaAgent is able to successfully complete task planning (TP), subtask grounding (SG), code generation (CG), and task execution (TE) based on the user’s instruction in the physical environment. In our experiments, we consider the 100 language commands listed in Supplementary Note 7, and each command is implemented 100 times in our lab, where five participants are invited as the subjects, and each time the wireless router is placed at a different random but known location. As shown in Table 1, we can see that the metaAgent can achieve TE success rates of 90.2% and 72.3% for the simple and complex tasks, respectively. In order to study the importance of the specific choice of LLM, we evaluate the effects of different choices of LLMs on the metaAgent’s performance. While it is expected that the generative performance of the language model will improve with better language models, it is unclear how the LLM size influences the final success rate. As shown in Table 2, we choose to experimentally test our metaAgent with four LLMs (“gpt-3.5-turbo”, “gpt-4”, “glm-3” and “glm-4”). We performed these tests on the aforementioned 100 commands using the different LLMs; the results show that the “gpt-4” model is the most effective, reaching 85% success rate, while the other models achieve >70% success rate. This finding is particularly exciting because it reveals how an improvement in the language models translates to a similar improvement in the metaAgent’s performance. These results hint at a potential future where the fields of language processing and metaAgents can collaboratively improve each other and scale together.

**Table 2:** Results of performance analysis for different large language models

models	Success Rate (%)
gpt-3.5-turbo	78.2
gpt-4	85.1
glm-3	73.9
glm-4	81.7

### Experimental results for long-horizon human-robot interactions

**Fig. 4:**
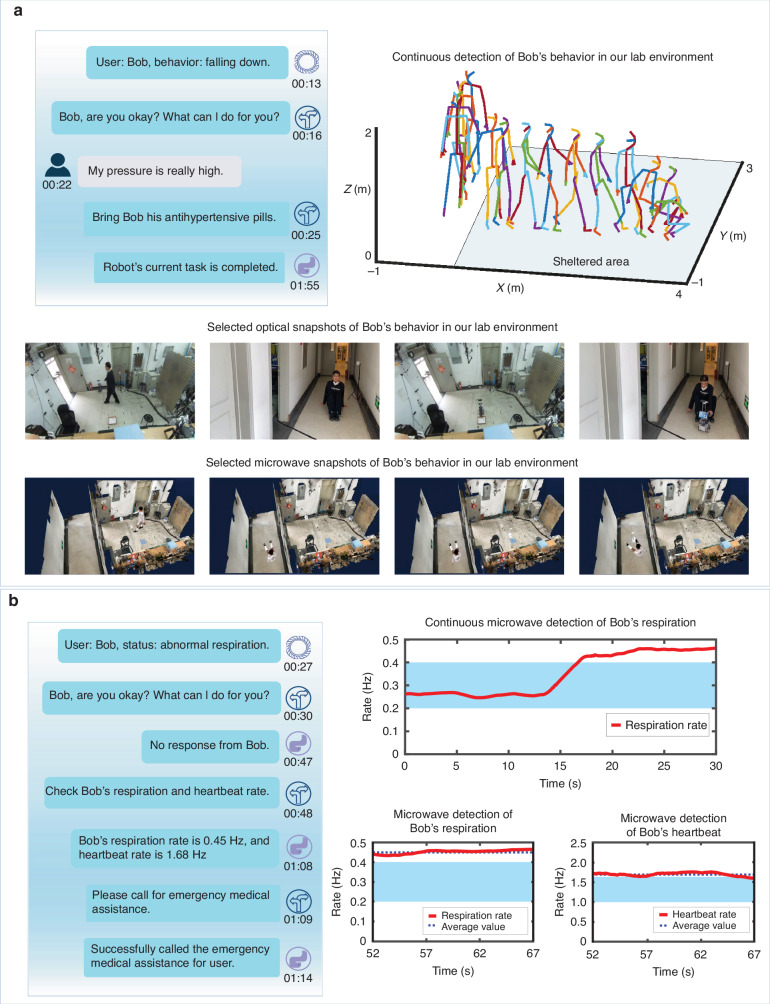
Two experimental results of long-horizon human-robot interactions. **a** The metaAgent recognizes the accidental fall of a user, and initializes communication with the user to help them out of danger based on their language instructions. (upper left) Excerpt of the protocol of communications between the human user and the metaAgent. Herein, the outputs of different domain experts in natural language are seen. (upper right) A sequence of reconstructed 3D human skeletons, based on microwave reconstruction in the sheltered region for privacy preservation, and based on an optical camera otherwise. (bottom) Some photographic snapshots of the robot. More details have been recorded in **Video S1**. **b** The metaAgent proactively helps the subject out of danger by checking the subject’s vital signs and contacting a medical expert when it determines that the subject has an emergency and does not communicate with the metaAgent. (left) Excerpt of the protocol of communications between the human and the metaAgent. (right) Some results of the human-robot interactions: (i) Recognition of the abnormal behavior based on a 30 s-long sample of Bob’s breathing rates, (ii) communicate with the subject, but fails to get the response, (iii) proactively monitor the breathing and heartbeat rates of subject, (iv) call the emergence number or human intervene for helping the subject out of the danger. More details have been recorded in Video S2

Finally, we consider a more realistic scenario to demonstrate the metaAgent’s capability in the realm of long-horizon human-robot interactions, where the metaAgent serves as a personal life assistant, autonomously and continuously responding to the live feedback of an evolving environment and the user’s requests. Selected experimental results are reported in Fig. 4, and more results have been recorded in Supplementary Videos 1 and 2. These results demonstrate that our metaAgent is capable of continuously monitoring the ‘general’ requests from the user, and making dynamically executable plans that consider the real-time context of the surroundings. Here, we mean by ‘general’ that user requests can be expressed in natural language, body language or vital signs. In previous sections, we had limited ourselves to requests in natural language. However, body language and vital signs are additional important expressions of user requests or needs. For instance, once the metaAgent perceives the user’s behavior to be unusual, such as an accidental fall or abnormal respiration, the metaAgent can rapidly make decisions and help the user out of danger through continuous interactions with the user.

We first consider a moderately complex case (see Fig. 4a and Video S1), where the metaAgent recognizes that a user accidently falls; the metaAgent then initializes communications with the user and helps the user out of danger following the user’s language instructions. The results demonstrate that the metaAgent, as a family life assistant, exhibits human-level performance, as argued below. First, the metaAgent can understand the intention that the user has asked for something by saying ‘my pressure is really high’, and then guide a mobile robot to bring antihypertensive drugs to the user. In other words, the metaAgent can recognize an abnormal feature of the subject in the observed real-time context. Second, the metaAgent is capable of continuously and autonomously monitoring the user in a complex indoor environment. Interestingly, the metaAgent can actively invoke a microwave sensor to implement a non-line-of-sight sensing task when the subject is outside the visually monitorable scene, for instance, when the user walks behind an opaque wall. This is also really important in scenarios requiring strict privacy preservation such as in a wash room. It can be observed that the metaAgent can perceive what the human cannot see by leveraging unique features of microwave radiation: independence of lighting conditions and penetration of visually opaque layers. In this sense, the metaAgent is capable of outperforming humans. In order to further evaluate the metaAgent’s performance, we have conducted the above-described experiments 50 times in our laboratory environment, and corresponding experimental results have been summarized in Table S1, where ten participants were invited to act according to the above scenario, and they randomly implemented the gesture of falling down somewhere in our laboratory. We observe that the metaAgent can achieve a planning success rate of 84% and an execution success rate of 74%, indicating that the metaAgent can accomplish the intended tasks in an uncertain real-world indoor environment.

Second, we consider the very complex scenario in which the metaAgent finds the subject in an emergency but fails to receive instructions from the subject. In contrast to the previous scenario, here, the metaAgent proactively helps the subject out of dangerous by examining the subject’s vital signs and asking for human intervention, as shown in Fig. 4b and Video S2. Note that the heartbeat and respiration are detected by SPM-based microwave sensing. In contrast to the aforementioned examples based on instructions in natural language, the present case is more challenging because it requires the metaAgent to possess the capability of autonomous planning based on the real-time environmental cues. Besides, the metaAgent is required to understand user needs based on the observed context, and the metaAgent must self-plan many steps without error, including the robot’s navigation. Furthermore, the metaAgent must understand chronology and history, from which it can recognize the subject’s situation, synthesize a brief report on the subject, and call an emergence number. Table S2 reports the statistical performance of the metaAgent over 50 experiments in our laboratory with different settings, similar to above, but the participants are asked to act with slower or faster respiration. It can be observed from these results that the metaAgent can achieve a planning success rate of 80%, a detection accuracy of human respiration of 90%, and an execution success rate of 78%.

## Discussion

To summarize, we presented and experimentally prototyped the concept of an EM metamaterial agent reasoning in natural language. Whereas previously reported metamaterials were realizing a pre-defined functionality as proxies of a human operator, we have conceived an autonomous metamaterial entity that reasons in natural language to perceive its environment, and to plan and execute its actions including programmable-metasurface-based EM wave manipulations for sensing and communications. Our experiments in the context of ambient-assisted living demonstrated our metaAgent’s capability to understand a variety of environmental cues (spoken instructions, body language, vital signs), to make long-horizon plans involving interactions with robots and humans, and to successfully execute the plans based on the available hardware and EM wave manipulation skills. Remarkably, the capabilities of the metaAgent even exceed those of a human assistant because of the metaAgent’s microwave perception that can sense through visually opaque layers and around corners. Our metaAgent’s reasoning in natural language was facilitated by a multi-agent discussion of various domain experts, each based on an LLM provided with a context demonstration. We observed that deploying more recent LLMs with more parameters yields a higher success rate of the metaAgent, which implies that the metaAgent will be able to harness the expected further rapid progress on LLMs in the coming years. Looking forward, it will be important to identify efficient techniques for scaling up the memory and action libraries while maintaining the metaAgent’s ability to efficiently formulate policies. One exciting route may be to guide the metaAgent to autonomously learn new skills, akin to children learning new skills under a teacher’s guidance at school.

## Materials and methods

### Programmable metasurfaces

Our metaAgent has access to three programmable metasurfaces operating in frequency bands centered on 2.4 GHz, 5.5 GHz and 9.7 GHz. Here, we briefly describe these programmable metasurfaces; more details have been provided in Supplementary Note 2. The 2.4 GHz meta-atom features two substrate layers: the top substrate is F4B with a relative permittivity of 2.55 and a loss tangent of 0.0019, and the bottom substrate is FR4. A PIN diode (SMP1345-079LF) is integrated into the top square patch and connected to the ground plane via a hole. An RF choke with inductance *L* = 33 nH is used to suppress the AC coupling to ground. The phase change can be accomplished by switching the external DC voltage applied to the PIN diode from 12 V to 0 V. The whole programmable metasurface is electronically controlled with an FPGA-based Micro-Control-Unit (MCU). To achieve the real-time and flexible control of 768 PIN diodes, an MCU with size of 90 × 90 mm^2^ is designed and assembled on the upper rear of the metasurface. In our work, the adopted CLK is 50 MHz, and the switching time of PIN diode is about 2.5 us each cycle. Each metasurface panel is equipped with eight 8-bit shift registers (SN74LV595APW), and groups of 8 PIN diodes share the same shift register. We remark that the proposed control strategy can be readily extended to more PIN diodes by concatenating more metasurface panels, allowing adjustable rearrangement of metasurface panels to meet various needs. The 5.5 GHz meta-atom has the same structure as the 2.4 GHz except that it scaled down in size. The 9.7 GHz meta-atom has three substrate layers: the top substrate is Taconic TLX-8 with a relative permittivity of 2.55, and the middle and bottom substrates are FR4. A MADP-000907-14020x PIN diode is integrated into the top square patch of the 9.7 GHz meta-atom. As opposed to the 2.4 GHz meta-atom, the RF choke inductance is replaced with a fan-shaped microstrip line and placed at the bottom of the meta-atom.

### Sensing expert

The sensing expert continuously takes multi-modal data as input, including sounds recorded by microphones, images recorded by cameras, text input via a keyboard and microwave signals captured by software defined radios, and outputs the semantic result for the planning expert. The sensing expert selects the appropriate deep neural networks or other signal processing methods to analyze and understand the inputs. For image and sound processing, models that were pre-trained on large-scale datasets are utilized. For instance, Xunfei API recognizes speaker identities and speech content, ZED SDK realizes multi-camera fusion for human keypoint detection. The microwave sensing is based on multiple programmable metasurfaces operating in different frequency bands which can be used to realize diverse sensing tasks (skeleton keypoint detection, human localization, behavior recognition, breathing and heartbeat monitoring). The microwave sensing uses the programmable metasurfaces to probe the environment and interprets the measured signals using artificial neural networks trained via supervised learning, as detailed in Supplementary Note 8. Note that these sensing algorithms are also shared with the coding expert. Microwave sensing complements optical sensing when the latter is ineffective (e.g., around corners or behind opaque layers) or unavailable (e.g., due to privacy concerns). Additional details about the sensing expert are also provided in Supplementary Note 3. The sensing expert synthesizes their understanding of the multi-modal input data in natural language and passes it on to the planning expert.

### Planning expert

The planning expert, drawing on knowledge provided in the memory module, analyzes the natural-language input from the sensing expert or the coding expert, and decomposes it into a series of feasible subtasks. The list of hierarchical subroutines to be executed is then passed on in natural language to the grounding expert. The planning expert is based on an LLM provided with a few context demonstrations, as detailed in Supplementary Note 4. In addition, the planning expert improves based on human feedback stored in the memory library.

### Grounding expert

The grounding expert receives the planning expert’s list of subtasks and assigns suitable action functions and associated devices for each subtask. Each action function corresponds to one of metaAgent’s EM wave manipulation skills. As detailed in the Methods, an action function may require the determination of parameters such as the coordinates of a human or an object, which may be accomplished by accessing sensor data or actively invoking a particular sensor. Once any pending parameters are determined, the action function may involve the manipulation of actuators such as robotic entities. Action functions and involved devices are stored in the memory module which can be updated to add new skills or modify the details of existing skills. The grounding expert is based on an LLM provided with a few context demonstrations, as detailed in Supplementary Note 5.

### Coding expert

The coding expert takes as input the triplet of intended goal, required action function and devices in natural language. In particular, the coding expert checks whether the various required subtasks can be executed in parallel or require sequential execution. Then, the coding expert writes python code that runs on the host computer using the known functions. The coding export produces two types of outputs in natural language: one ‘external’ output for communication with the human user via the chat tool, and one ‘internal’ output for consideration by the planning and grounding experts. The ‘internal’ output is a crucial part of the metaAgent’s reasoning in natural language via the multi-agent discussion on which the metaAgent’s autonomous self-organization skills build. The coding expert is based on an LLM provided with a few context demonstrations, as detailed in Supplementary Note 6.

### Action Library

The Action library stores the various actions that can be performed by the SPM for the metaAgent to decompose and assign tasks. In addition, the name of the executable device for each specific action performed is also provided. The action module follows two steps. The first step is to select an action function from the Action Library. This action function indexes a specific operations flow. Meanwhile, the parameters of this action function might need to be determined. For example, when moveToPosition() is selected, there are three undetermined parameters, namely the coordinates (x, y, z) of the desired position. This flow contains access to sensor results and manipulations of actuators, as well as actively invoking a particular sensor. After the flow ends, the parameters of the action function will be determined, and then the final action operation will be executed.

### Memory Library

The memory library is utilized to store a priori information about the physical environment, but it also empowers the metaAgent to assimilate knowledge progressively. For instance, the memory library also stores the history of user interactions as well as past perceptions of the environment. Thereby, the metaAgent is capable of swiftly evaluating alterations in the environment by contrasting the latest captured image with preceding ones. The metaAgent primarily exploits vector databases for the efficient description and retrieval of memories, thus enabling the fetching of pertinent memories from extensive repositories by amalgamating similarity searches with additional parameters.

## Supplementary information

Supplementary Information

Supplementary Video 1

Supplementary Video 2

## Data Availability

The data that support the findings of this study are available from L. L. upon request.
