# Supplementary material for: Electromagnetic metamaterial agent
Source: Light Sci Appl. 2025 Jan 1;14:12. doi: 10.1038/s41377-024-01678-w (PMC11688436; doi:10.1038/s41377-024-01678-w)
Supplement: Supplementary file 1 — Supplementary Information [file 41377_2024_1678_MOESM1_ESM.docx]

**Supplementary Information for**

**Electromagnetic metamaterial agent**

Shengguo Hu^1+^, Mingyi Li^1+^, Jiawen Xu^1+^, Hongrui Zhang^1+^, Shanghang Zhang^2^, Tie Jun Cui^3,4^, Philipp del Hougne^5^, and Lianlin Li^1, 4^

^1^ State Key Laboratory of Advanced Optical Communication Systems and Networks,

School of Electronics, Peking University, Beijing 100871, China

^2^ National Key Laboratory for Multimedia Information Processing, School of Computer Science, Peking University, Beijing 100871, China

^3^ State Key Laboratory of Millimeter Waves, Southeast University, Nanjing 210096, China

^4^ Pazhou Laboratory (Huangpu), Guangzhou, Guangdong 510555, China

^5^ Univ Rennes, CNRS, IETR - UMR 6164, F-35000 Rennes, France

^+^ Equal contribution.

**Video S1:** **Supporting video for Figure 4a.**

**Video S2:** **Supporting video for Figure 4b.**

**Table S1: Experimental results of long-horizon human-robot interactions case 1.**

| Case 1 | Sensing expert | Planning expert | Grounding expert | Coding expert | Task execution |
| --- | --- | --- | --- | --- | --- |
| Success rate (%) | 98 | 84 | 92 | 90 | 74 |

**Table S2: Experimental results of long-horizon human-robot interactions case 2.**

| Case 2 | Sensing expert | Planning expert | Grounding expert | Coding expert | Task execution |
| --- | --- | --- | --- | --- | --- |
| Success rate (%) | 90 | 80 | 98 | 96 | 78 |

**Supplementary Note 1.** **System configuration and operation pipeline of metaAgent.**

Here, we introduce the components and operation pipeline of metaAgent in detail. First, we introduce the system configuration of metaAgent. As shown in **Figure S1**, the metaAgent comprises two components: a cerebellum centered on semantically programmable metasurfaces (SPMs) for executing specific EM manipulation tasks, and another part, the cerebrum, based on large-capacity foundation models (LFMs), utilized for formulating high-level strategies at the human level. For the cerebrum part, it mainly consists of four domain experts. They are sensing expert for external environment perception, planning expert for task planning and decomposition, grounding expert for action selection and device assignment and coding expert for code generation. Among them, the sensing expert is designed by conventional deep neural networks, and the rest of the experts are based on modern large language model. In addition, we also equipped a memory module for the cerebrum to store data such as environmental prior knowledge, optional action skills, and historical interaction records. Here, the memory module consists of two main knowledge libraries, which are the memory library and the action library. Among them, the memory library contains the visual semantic map (VSM) and knowledge graph (KG) (**see** **Supplementary Note 10**) corresponding to the metaAgent's operating environment, as well as the historical data of user interactions, and the action library contains the action skills and optional devices possessed by the metaAgent, and provides detailed instructions for use by LLM-based experts, see more details in **Supplementary Note 11**. For the cerebellum part, it is mainly composed of two SPMs operating at 2.4 GHz and 5.5 GHz frequencies as the core, as well as a multitude of other modal sensing sensors, including a ZED2 camera, two Universal Software Radio Peripheral (USRP) devices with transceiver antennas, and a wireless microphone. SPMs operate on the electromagnetic environment by executing semantic coding patterns corresponding to a series of actions formulated for them by the cerebrum to interact with users, robots, and objects in the environment. As a result, the cerebrum could control the cerebellum to accomplish the corresponding actions, and then the cerebellum would feedback the collected results to the cerebrum.

**Figure S2** shows a map of our laboratory environment and the location of each sensor and the SPMs.

Next, we introduce the detailed operation pipeline of metaAgent. The operation pipeline of metaAgent is that the cerebrum formulates semantic coding patterns corresponding to a series of actions for the cerebellum based on the language instructions or other applicable instructions inputted by the user, and then the cerebellum executes the actions, thus accomplishing the user's instructions. As shown in **Figure S3**, the user can input instructions through voice, gesture, and text. First, the sensing expert transforms the user's speech instructions or other inputs along with the other modal data perceived into language task description in an annotated format for input to the planning expert. Then, the planning expert makes an abstract understanding of the task based on the memory module and decomposes the task into a series of sub-tasks that can be executed by the metaAgent based on the knowledge of the metaAgent's capabilities. Next, the grounding expert will select an appropriate action function and allocate the associated devices for each subtask based on the action library. Finally, the coding expert will output the sequence of semantic coding patterns corresponding to the selected SPM and give it to the executor for execution, based on the expected goal, action function, and device provided as inputs by the planning and grounding experts. See **Supplementary Note 3, 4, 5 and 6** for more detailed descriptions of the four experts. Here, in the subsequent experiments, the code generated by the coding expert is based on the python language. When the executor has completed executing the code it will output two types of results in verbal form: one for communicating with the user via microphone or chat tool, and the other for "internal" commands used by planning and grounding experts. If the task is completed, the metaAgent will end the task. If an error is reported in the execution of the code, the executor will send the error information back to the coding expert, who will analyze it and generate a new correct code. In addition, we have also incorporated a human feedback mechanism so that when the metaAgent makes an incorrect task decomposition or wrong action selection, the user can participate in a multi-expert discussion to guide the metaAgent through the subsequent planning tasks, and store the useful feedback in the memory library for the metaAgent's subsequent reference.


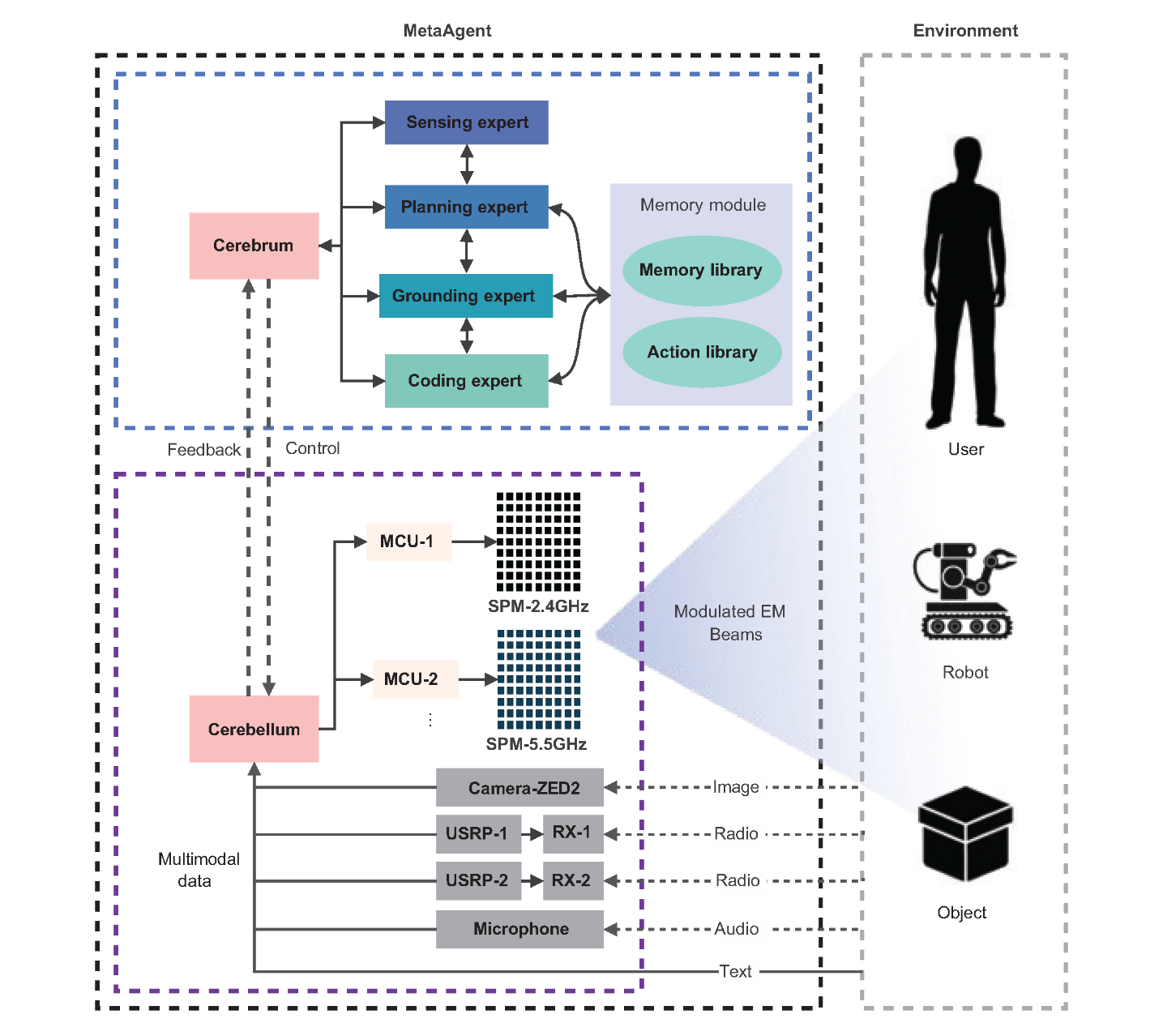


**Figure S1** | The system configuration of the metaAgent.


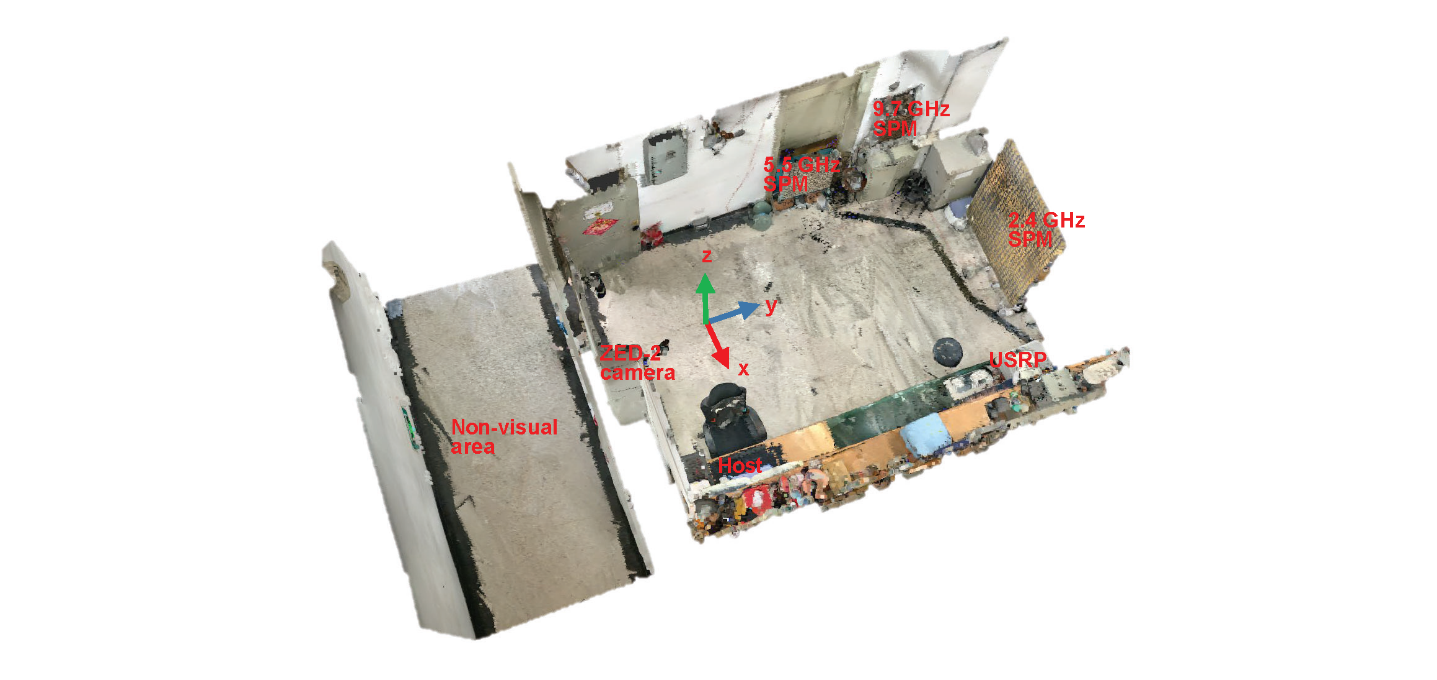


**Figure S2** | The lab environment map.


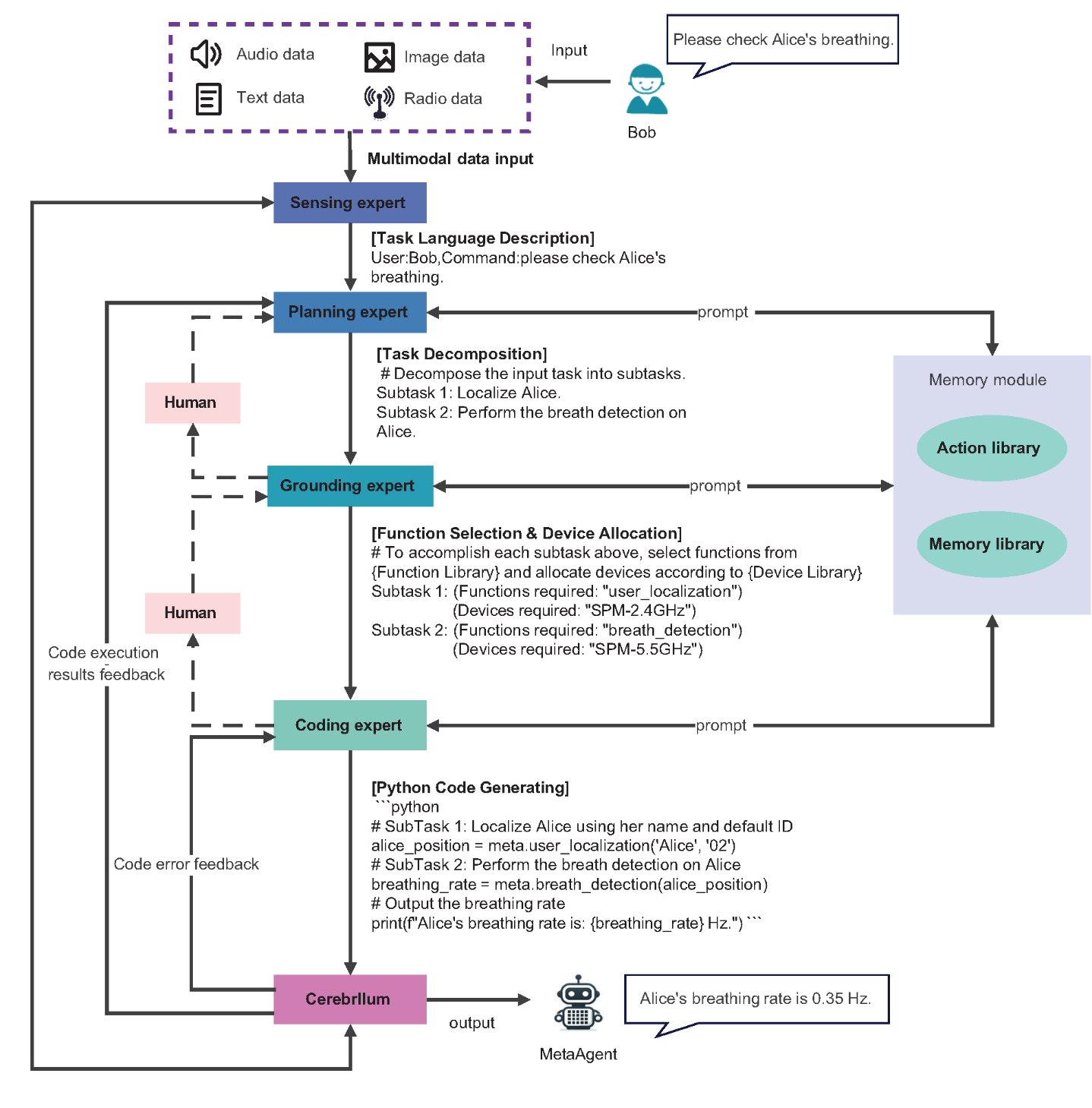


**Figure S3** | The operation pipeline of metaAgent.

**Supplementary Note 2. Semantically programmable metasurfaces-SPMs**

The Semantic Programmable Metasurface (SPM) is based on the programmable coding metasurface design. We mapped high-level language instructions to conventional binary digit coding sequences of programmable metasurfaces to obtain semantic coding patterns for SPM. Thus, the semantic control coding patterns of the SPM reflect the high-level language instructions entered in the deployment environment. Next, we first introduce the programmable metasurfaces in detail and then introduce the modified Gerchberg-Saxton (G-S) algorithm.

**(1) Programmable metasurfaces.**

Our system applies 1-bit metasurfaces operating in three frequency band centered on 2.4 GHz, 5.5 GHz and 9.7 GHz^[1]^. The models of the metasurface units (i.e. meta-atoms) and their electromagnetic responses are shown in **Figure S4**. The detailed parameters of the metasurfaces and meta-atoms are shown in **Table S3**. The structure of each meta-atom mainly consists of a rectangular metal resonant patch, a phase bias line, a dielectric substrate and a metal reflector plate. A PIN diode is integrated between the metal patch and the phase bias line of each meta-atom and connected to the ground plane by a metal through-hole. For the 2.4 GHz and 5.5 GHz meta-atom, an RF choke with inductance L = 33 nH is used to suppress the AC coupling to ground. For the 9.7 GHz meta-atom, the RF choke inductance is replaced with a fan-shaped microstrip line placed at the bottom of the meta-atom. The "on" and "off" states of the PIN diode can be controlled by adjusting the voltage across the PIN diode to either 12 V or 0 V, thus reversing the phase of the reflected electromagnetic wave by 180 °. These two states are encoded as 1 and 0. Each metasurface subpanel consists of 8×8 identical meta-atoms, and is controlled by a Micro-Control-Unit (MCU) with size of 90×90 mm^2^, while each metasurface consists of 3×4 subpanel linked together, so that each metasurface contains 768 controllable meta-atoms, and we refer to the PIN diode states of these meta-atoms as a control coding pattern. Each metasurface subpanel is equipped with eight 8-bit shift registers (SN74LV595APW), and every 8 PIN diodes share the same shift register. The control coding pattern is configured in the FPGA by the host computer and output to each MCU through the GPIO port of the FPGA via serial-to-parallel conversion, and the MCU outputs the corresponding voltage to the PIN diode of the meta-atoms. In our work, the adopted CLK is 50 MHz, and the switching time of the PIN diode is about 2.5 us each cycle.


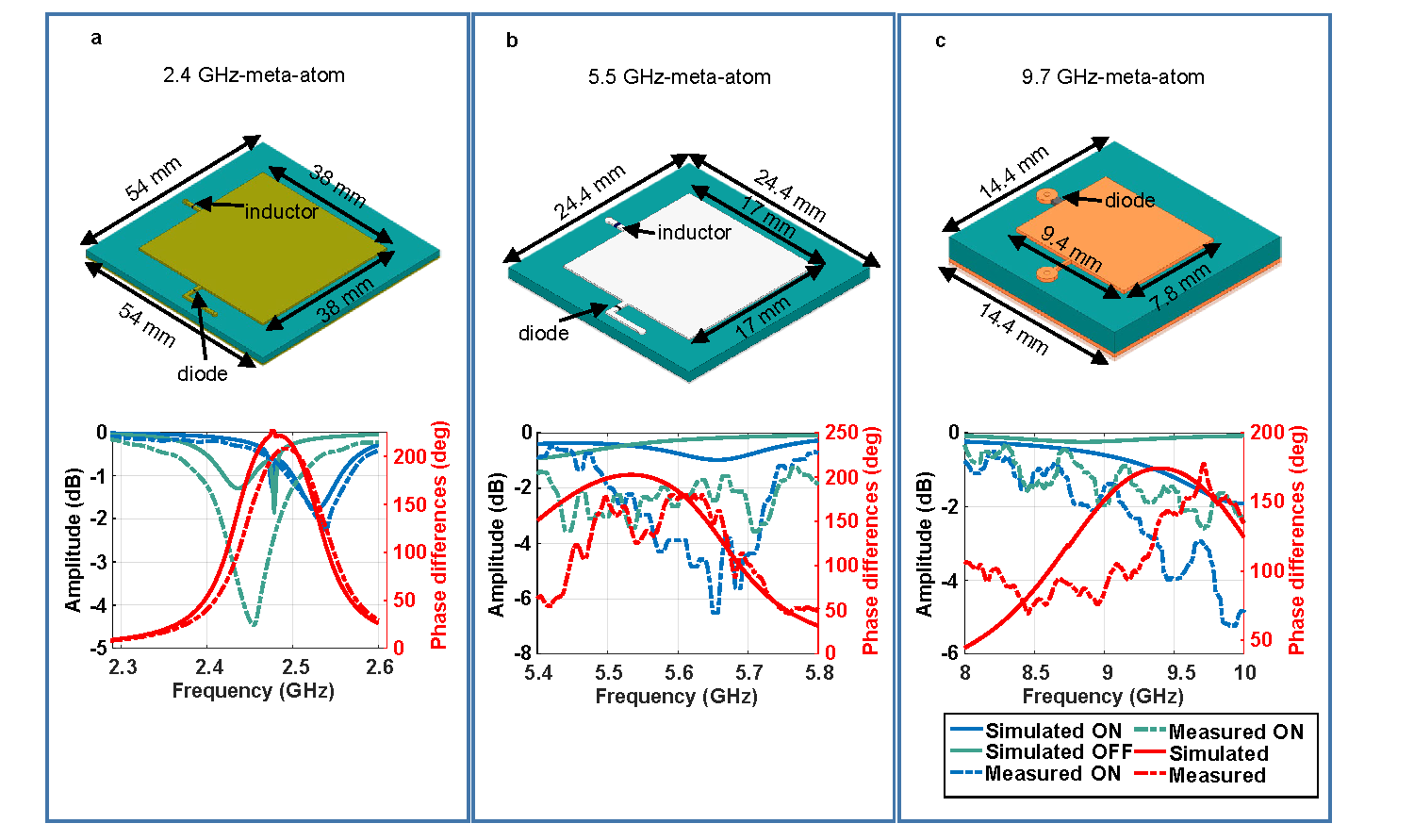


**Figure S4** | The models and electromagnetic responses of 2.4 GHz, 5.5 GHz, 9.7 GHz meta-atoms. (**a**) 2.4 GHz meta-atom. (**b**) 5.5 GHz meta-atom (**c**) 9.7 GHz meta-atom

**Table S3** | The detailed parameters of the metasurfaces and meta-atoms

| **parameters** | **metasurfaces** | | |
| --- | --- | --- | --- |
|  | **2.4 GHz** | **5.5 GHz** | **9.7 GHz** |
| Meta-atom array | 32×24 | 32×24 | 32×24 |
| Array size | 1.7 m×1.3 m | 0.78 m×0.59 m | 0.46 m×0.35 m |
| Meta-atom size | 54 mm×54 mm | 24.4 mm×24.4 mm | 14.4 mm×14.4 mm |
| PIN type | SMP1345-079LF | SMP1345-079 LF | MADP-000907-14020x |
| Top substrate | F4B | F4B | Taconic TLX-8 |
| Middle substrate | - | - | FR4 |
| Bottom substrate | FR4 | FR4 | FR4 |

**(2) The modified Gerchberg-Saxton (G-S) algorithm.**

Different control coding patterns lead to different EM radiation patterns. In order to design the control coding pattern according to the desired EM radiation pattern, we use a modified Gerchberg-Saxton (G-S) algorithm, which is centered on optimizing the control coding pattern of the metasurface so that the scattered field obtained from the forward computation $\boldsymbol{E}$ is as close as possible to the target scattered field $\boldsymbol{E}_{\mathrm{goal}}$. We consider that the scattered field of the metasurface is formed by the excitation of surface currents on each meta-atom. Supposing that the 0 and 1 state of the meta-atom correspond to the surface currents $\boldsymbol{J}_{0}$ and $\boldsymbol{J}_{1}$, and the scattered field $\boldsymbol{E}$ can be calculated by the Green's function based on the surface current matrix of the metasurface array $\boldsymbol{J}$, i.e.$\boldsymbol{E}$ = $\boldsymbol{A}\boldsymbol{J}$, where $\boldsymbol{A}$ denotes the mapping matrix. Similarly, there is its inverse transformation$\boldsymbol{J} = \boldsymbol{A}^{-\mathbf{1}}\boldsymbol{E}$. In order to avoid the effect of the pathology matrix, the inverse transformation is corrected as$\boldsymbol{J}={{(\boldsymbol{A}^{'}\boldsymbol{A}+\gamma\mathbf{I})}^{+}\boldsymbol{A}}^{'}\boldsymbol{E}$. The workflow of the improved G-S algorithm is shown in **Supplementary Table 4**: (1) Set $\boldsymbol{E}$ as $\boldsymbol{E}_{\mathrm{goal}}$. (2) Calculate $\boldsymbol{J}$, according to the inverse transformation matrix. $\boldsymbol{J}={{(\boldsymbol{A}^{'}\boldsymbol{A}+\gamma\mathbf{I})}^{+}\boldsymbol{A}}^{'}\boldsymbol{E}$. (3) Determine the control coding pattern of the metasurface $\mathcal{C}$ based on the phase of $\boldsymbol{J}$ , specifically, the (i,j) th meta-atom is coded as 0 when the phase of $\boldsymbol{J}(i,j)$the is between 0 and $\pi$, otherwise it is coded as 1. (4) Calculate the scattered field $\boldsymbol{E}$ based on the updated control coding pattern $\mathcal{C}$ . (5) Update $\boldsymbol{E}$ by keeping its phase unchanged and replacing the amplitude with the amplitude of $\boldsymbol{E}_{\mathrm{goal}}$ . (6) Determine whether the difference between $\boldsymbol{E}$ and $\boldsymbol{E}_{\mathrm{goal}}$ satisfies the requirement, if it does, output the control coding pattern of the metasurface $\mathcal{C}$, if it does not, re-execute step (2).

**Table S4** | The modified Gerchberg-Saxton (G-S) algorithm.

| Initialize system parameters: mapping matrix ***A***, target scattered field $\boldsymbol{E}_{\text{goal}\text{ }}$.   1. $\boldsymbol{E}_{0}=\boldsymbol{E}_{\mathrm{goal}}$ 2. $\mathrm{WHILE}\left( \left\vert\boldsymbol{E}_{k}-\boldsymbol{E}_{\text{goal }} \right\vert>\epsilon\text{ or }k=0 \right)\boldsymbol{J}_{k}=\left( \mathbf{A}^{'}\mathbf{A}+\gamma\mathbf{I} \right)^{+}\boldsymbol{A}^{'}\boldsymbol{E}_{k}$ 3. Traversing each meta-atom, i.e., for any $i\in\{1,2,\ldots,32\},j\in\{1,2,\ldots,24\}$, the unit equivalent surface current at coordinates $\left( i,j \right)$ is determined to be   $\boldsymbol{J}^{'}\left( i,j \right)=\left\{ \begin{aligned} &\boldsymbol{J}_{0},\text{ }\text{if }0\leq\mathrm{angle}\left( \boldsymbol{J}_{k}\left( i,j \right) \right)<\pi\\ &\boldsymbol{J}_{1},\text{ }\text{if }\pi\leq\mathrm{angle}\left( \boldsymbol{J}_{k}\left( i,j \right) \right)<2\pi\end{aligned} \right.$  $\mathcal{C}\left( i,j \right)=\left\{ \begin{aligned} &0,\text{ if }\boldsymbol{J}^{'}\left( i,j \right) = \boldsymbol{J}_{0} \\ &&1, \mathrm{if}\boldsymbol{J}^{'}\left( i,j \right) = \boldsymbol{J}_{1} \end{aligned} \right.$   1. $\boldsymbol{E}_{k}=\mathbf{A}\boldsymbol{J}^{'}$ 2. $\boldsymbol{E}_{k+1}=\left\vert\boldsymbol{E}_{\text{goal }} \right\vert e^{j\cdot\text{ angle }\left( \boldsymbol{E}_{k} \right)}$ 3. $k=k+1$   END WHILE |
| --- |

**Supplementary Note 3. Sensing expert**

The sensing expert is responsible for collecting and analyzing multi-modal data from the environment and delivering the sensing results to the planning expert in text form. As depicted in **Figure S5**, it continuously receives data in various modalities such as audio, radio, visual and text from multiple sensors and selects the appropriate deep neural network or signal processing method to analyze and understand these data depending on the task. The models for processing sound and image are typically pre-trained on large-scale datasets and can be integrated into the sensing expert systems through local deployment or API invocation. We have deployed microphones in several areas of the lab, and in terms of sound processing, we use Xunfei API to implement voiceprint recognition and speech recognition, which can recognize the identity of the speaker and the content of speech. We utilize multiple ZED 2 stereo cameras to perceive optical images of the lab. The sensing expert invokes the ZED SDK to realize multi-camera fusion for skeleton keypoint, human localization and behavior recognition. Microwave sensing complements optical sensing when the latter is ineffective. Microwave sensing is based on multiple multi-frequency intelligent metasurfaces, which can realize diverse sensing tasks, including skeleton keypoint detection, human localization, behavior recognition, breathing detection and heartbeat detection, for technical details refer to **Supplementary Note 8**. Here, considering the communication between the sensing expert and the other experts as well as the more flexible invocation of perceptual skills, we have configured an LLM on the output side for integrating the outputs of the various sensors into a semantic text. We specify a standard format for the output of sensing expert, i.e. User: {user_name}, Action: {user_action}, Status: {user_status}, Command: {user_command}. Here, the item is not output if there is no sensing data for that. The sensing expert continuously detects user behavior and health status in the physical environment, and as soon as a user anomaly or user input is detected, it outputs the results to the planning expert and shuts down the use of all the sensors to the coding expert until the end of the current task, when it will be activated again.


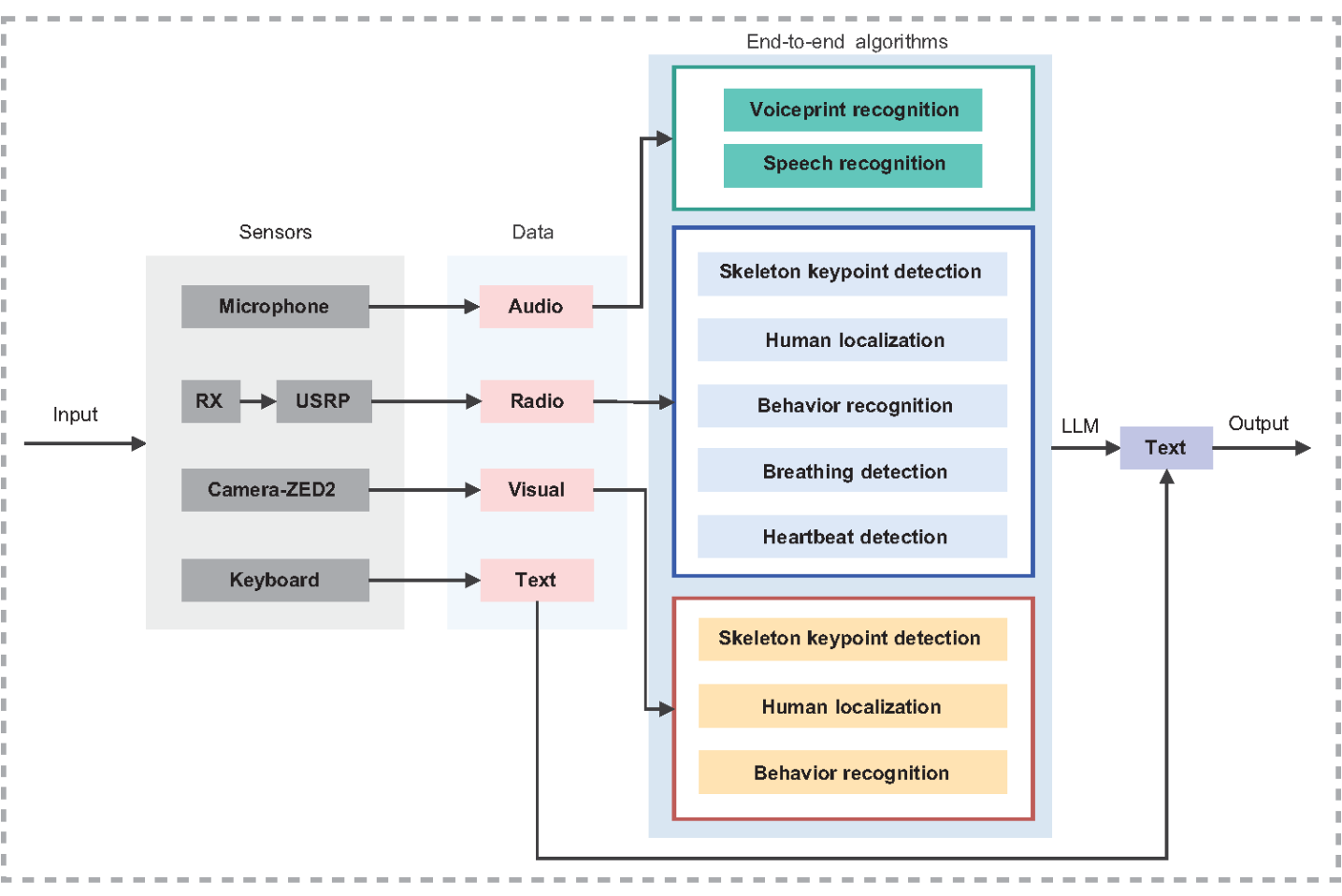


**Figure S5** | The workflow of the sensing expert.

**Supplementary Note 4. Planning expert**

The planning expert, as the central decision maker of the whole cerebrum, plays a key role in task comprehension and task planning. When the sensing expert communicates the collected linguistic task descriptions to the planning expert, the planning expert will first understand and analyze the input through the knowledge provided by the memory module and decompose the task into a series of subtasks. Typically, user instructions are ambiguous and intricate in nature in most scenarios. Hence, the planning specialist must utilize a comprehensive language model to break down the directive into a sequence of feasible sub-tasks considering the MetaAgent's abilities and the prevailing circumstances, while also establishing the interdependencies and the sequential execution of these sub-tasks. To facilitate efficient task planning with the extensive language model, a chain-of-thought-based framework is employed in prompt formulation, supplemented by a few illustrative instances. The prompt project of the planning expert used is given in **Table S5**. By injecting a few demo cases into the prompts, this few-shot learning approach allows the planning expert to better understand the intent and criteria of task planning. Here, each demo case is a standard formatted input and output, which effectively helps the planning expert to understand the logic between tasks and consequently determine the dependencies and execution order between subtasks. In addition, after the planning expert completes the task decomposition, we also introduced a human feedback mechanism, which further helps the planning expert to correct the wrong planning results. At the same time, the result of the human feedback is stored in the memory library to guide it for other subsequent planning tasks.

**Table S5** | The prompt project of the planning expert.

#01 You are a planning expert. You need to make autonomous decisions based on the outputs of sensing expert and executor.

If input is {User} and {Command}, you need to decompose the user commands into a series of sub-tasks based on the contents of the {Memory Library} and {Action Library} that I have provided for you.

If input is {User} and {Action} or {Status}, you need to determine if the user's behavior or state is abnormal,and if so, you need to immediately engage the user in a voice interaction to seek whether the user needs help.

If input {Command: No response from user}, you need to check user's respiration and heartbeat rate.

If user's respiration or heartbeat rates rates are again detected as abnormal, call for emergency medical assistance immediately.

If there is no input from the sensing expert and executor, reply "END" to end the task.

#02 Task decomposition steps must be based on the "function library" and cannot simplify or add tasks that cannot be accomplished.

#03 It is important to read through the contents of the {common-sense Library} before you perform a task decomposition, which will make some of the task decomposition rules more clear to you.

#04 You need to describe the dependencies between the subtasks.

#05 Do not make any assumptions.

#06 Must output the results in the format of the {examples}.

<<Action_library>>

<<Memory_library>>

Here provide several {examples} for you:

"""

(Example 1):

Input:

User:Alice,Command:Perform behavior recognition on Bob.

Output:

Task: Perform behavior recognition on Bob.

[Task Decomposition]

# To perform behavior recognition on Bob, we need to use the 'behavior_recognition' function. This function requires the user's position as input, which can be obtained using the 'user_localization' function.

# Decompose the input task into subtasks.

Subtask 1: Localize Bob.

(Subtask required: None)

Subtask 2: Recognize Bob's behavior.

(Subtask required: "Subtask 1")

(Example 2):

Input:

User:Bob,Command:Please enhance the signals at the table.

Output:

Task: Enhance the signals at the table.

[Task Decomposition]

# Enhancing the signal at the table requires the 'communication_link_enhance' function. For 'communication_link_enhance', we need two parameters: the source of the signal, which is the 'router', and the target location, which is the 'table'. The 'router' can be found in {Environment Library} using the function 'search_object_from_map', and the 'table's location can also be found in the same way since it exists in {Environment Library}.

# Decompose the input task into subtasks.

Subtask 1: Get the router's location from the environment library.

(Subtask required: None)

Subtask 2: Get the table's location from the environment library.

(Subtask required: None)

Subtask 3: Use the router to enhance the signal for the table.

(Subtask required: "Subtask 1","Subtask 2")

(Example 3):

Input:

User: Alice, Action: falling down.

Output:

Task:Alice's behavior is abnormal. Ask Alice if she needs help.

Subtask 1: Start a voice interaction with Alice to find out if she needs help.

(Subtask required: None)

(Example 4):

Input:

User:Alice, Status: {respiration}:27.

Output:

Task:Alice's respiration is abnormal. Ask Alice if she needs help.

Subtask 1: Start a voice interaction with Alice to find out if she needs help.

(Subtask required: None)

(Example 5):

Input:

User:Alice, Command: No response from user.

Output:

Task: Check user's respiration and heartbeat.

Subtask 1: Localize Alice to determine her current position for relevant detections.

(Subtask required: None)

Subtask 2: Perform breath detection for Alice.

(Subtask required: "Subtask 1")

Subtask 3: Perform heartbeat detection for Alice.

(Subtask required: "Subtask 1")

"""

**Supplementary Note 5.** **Grounding expert**

The primary role of the grounding expert is to facilitate the integration of the task at hand with the physical environment entity through active engagement with the surroundings, thereby enhancing the MetaAgent's capacity for adaptable and intelligent interaction with the physical environment. After the planning expert has decomposed the task into a sequence of subtasks, the grounding expert will allocate the appropriate action functions and devices to each subtask. To do this, the grounding expert first needs to obtain the devices available in the environment and the function type of each device. Here, the action functions used in our experiments are stored in the memory module, and each action function corresponds to a skill of the metaAgent. In addition, the user has the flexibility to add new skills or modify inappropriate skills according to their actual needs, the details of the specific skills are described in the **Supplementary Note 8**. Here, we also provide a few demonstration examples for grounding expert to standardize the output in the prompt design.The prompt project of the grounding expert used is given in **Table S6**.

**Table S6** | The prompt project of the grounding expert.

#01 You are a grounding expert and you need to allocate the appropriate action and device to each of the sub-tasks based on a series of sub-tasks provided to you by the planning expert.

#02 Each subtask can only select one function and assign one device.

#03 Do not make any assumptions.

#03 The selected function and device must be existing in {Function Library} and {Device Library}.

#04 Must output the results in the format of the {examples}.

<<Action_library>>

Here provide several {examples} for you:

"""

(Example 1):

input:

[Task Decomposition]

# To perform behavior recognition on Bob, we need to use the 'behavior_recognition' function. This function requires the user's position as input, which can be obtained using the 'user_localization' function.

# Decompose the input task into subtasks.

Subtask 1: Localize Bob.

(Subtask required: None)

Subtask 2: Recognize Bob's behavior.

(Subtask required: "Subtask 1")

output:

[Action & Device Allocation]

# To accomplish each subtask above, select functions from {Function Library} and allocate devices according to {Device Library}

Subtask 1: Localize Bob.

(Functions required: "user_localization")

(Devices required: "SPM_2.4GHz")

(Subtask required: None)

Subtask 2: Recognize Bob's behavior.

(Functions required: "behavior_recognition")

(Devices required: "SPM_5.5GHz")

(Subtask required: "Subtask 2")

(Example 2):

input:

[Task Decomposition]

# Enhancing the signal at the table requires the 'communication_link_enhance' function. For 'communication_link_enhance', we need two parameters: the source of the signal, which is the 'router', and the target location, which is the 'table'. The 'router' can be found in {Environment Library} using the function 'search_object_from_map', and the 'table's location can also be found in the same way since it exists in {Environment Library}.

# Decompose the input task into subtasks.

Subtask 1: Get the router's location from the environment library.

(Subtask required: None)

Subtask 2: Get the table's location from the environment library.

(Subtask required: None)

Subtask 3: Use the router to enhance the signal for the table.

(Subtask required: "Subtask 1","Subtask 2")

output:

[Action & Device Allocation]

# To accomplish each subtask above, select functions from {Function Library} and allocate devices according to {Device Library}

Subtask 1: Get the router's location from the environment library.

(Functions required: "search_object_from_KG_VSM")

(Devices required: "host")

(Subtask required: None)

Subtask 2: Get the table's location from the environment library.

(Functions required: "search_object_from_KG_VSM")

(Devices required: "host")

(Subtask required: None)

Subtask 3: Use the router to enhance the signal for the table.

(Functions required: "communication_link_enhance")

(Devices required: "metasurface_2.4GHz")

(Subtask required: "Subtask 1","Subtask 2")

"""

**Supplementary Note 6. Coding expert**

In the context of "coding-as-policy," the coding expert takes as input the triple of intended goal, action function, and device(s), and output a series of semantic coding patterns for the selected SPMs. Once all the subtasks have been allocated actions and the corresponding execution devices, it can then be handed over to the coding expert to write the program code for the required execution. First, before generating the code, the coding expert needs to analyze and think about the results provided by the planning expert and the grounding expert in order to determine the execution order of the subtasks. For example, if sub-task 1 and sub-task 2 have no dependencies and sub-task 3 depends on the results of sub-tasks 1 and 2, we can generate code for sub-tasks 1 and 2 to execute in parallel. Subtask 3 is executed sequentially after tasks 1 and 2 have been executed. Here, we have encapsulated all the action functions that MetaAgent has into separate functions. The code expert only needs to write python code that runs on the host computer according to a strict specification for the use of the known functions. The prompt project of the coding expert used is given in **Table S7**. We have also given a few demonstration examples of code generation in the prompt project, which will help the coding expert to generate standardized code. When the code is generated, MetaAgent will automatically execute the code on the host device, and if the execution is successful, it will return the execution result of the corresponding command. If the MetaAgent makes an error while executing the code, the MetaAgent will re-call the coding expert and send the error back to the coding expert. It will analyze the type of error and regenerate the corrected code.

**Table S7** | The prompt project of the coding expert.

#01 You are a coding expert and you need to write python code based on the information provided to you by the grounding expert.

#02 You need to write task execution code based on sub-task dependencies and device occupancy. If there are no dependencies between tasks and the same device is used, you can write parallel execution code, otherwise, write sequential execution code.

#03 Do not import other Python library except threading.

#04 Do not declare additional variables.

#05 Do not make any assumptions.

#06 You must use the functions I provide in the {function Library}, you cannot define your own classes or functions without authorization

#07 Must generate python code based on the demo {examples} I gave you.

<<Action_library>>

<<Memory_library>>

Here provide several {examples} for you:

"""

(Example 1):

input:

[Action & Device Allocation]

# To accomplish each subtask above, select functions from {Function Library} and allocate devices according to {Device Library}

Subtask 1: Localize Bob.

(Functions required: "user_localization")

(Devices required: "SPM_2.4GHz")

(Subtask required: None)

Subtask 2: Recognize Bob's behavior.

(Functions required: "behavior_recognition")

(Devices required: "SPM_5.5GHz")

(Subtask required: "Subtask 2")

output:

[Thought]

# According to the result in [Action & Device Allocation],subtask 1 and 2 are dependent on each other sequentially. Subtask 2 cannot be executed before completing Subtask 1, as it requires the position of Bob from Subtask 1. Therefore, we should execute subtask 1 and subtask 2 in sequence.

[Python Code Generating]

```python

from meta_wrapper import *

meta = MetaWrapper()

print("Tasks Started")

# SubTask 1: Localize Bob.

bob_position = meta.user_localization('Bob')

# SubTask 2: Recognize Bob's behavior.

behavior = meta.behavior_recognition(bob_position)

print(f"Bob's behavior is recognized as: {behavior}.")

```

(Example 2):

input:

[Action & Device Allocation]

# To accomplish each subtask above, select functions from {Function Library} and allocate devices according to {Device Library}

Subtask 1: Get the router's location from the environment library.

(Functions required: "search_object_from_KG_VSM")

(Devices required: "host")

(Subtask required: None)

Subtask 2: Get the table's location from the environment library.

(Functions required: "search_object_from_KG_VSM")

(Devices required: "host")

(Subtask required: None)

Subtask 3: Use the router to enhance the signal for the table.

(Functions required: "communication_link_enhance")

(Devices required: "metasurface_2.4GHz")

(Subtask required: "Subtask 1","Subtask 2")

output:

[Thought]

# According to the result in [Action & Device Allocation], Subtask 1 and Subtask 2 cannot be executed in parallel since they do not depend on each other but require same devices. After obtaining the router and table's location, we use these locations in Subtask 3 to enhance the signal, which depends on the results of Subtask 1 and Subtask 2. Therefore, Subtask 3 should be executed after Subtask 1 and Subtask 2 are completed.

[Python Code Generating]

```python

from meta_wrapper import *

meta = MetaWrapper()

print("Tasks Started")

# Subtask 1: Get the router's location from the environment library.

router_position = meta.search_object_from_KG_VSM('router')

# Subtask 2: Get the table's location from the environment library.

table_position = meta.search_object_from_KG_VSM('table')

# executing SubTask 3

enhancement_success = meta.communication_link_enhance(router_position, table_position)

if enhancement_success:

print("The signal at the table has been successfully enhanced.")

else:

print("The signal enhancement at the table failed.")

```

**Supplementary Note 7. Set of language instructions for testing metaAgent at different levels of complexity**

In our experiments, we considered 100 language commands of varying complexity. In particular, we categorized tasks that require no more than 3 steps to complete as simple tasks and tasks that require more than 3 steps to complete as complex tasks. **Table S8** gives the 100 user instructions we tested, including some commands with the same meaning but different expressions, to test the linguistic flexibility of the metaAgent. For example, "Robot A, come to my location." or "Robot A, please see me.", or even "Robot A, come.", the user's command language is gradually blurred to evaluate the linguistic flexibility of the metaAgent.

**Table S8** | Set of language instructions.

| Simple instructions | 1. Where is the table?  2. Where is the box 1?  3. Where is Alice?  4. Please locate Bob.  5. Please locate Bob and Alice.  6. Where is my cell phone?  7. Where is Bob’s cell phone?  8. Where is robot A?  9. Please locate robot A and B.  10. Please locate the router.  11. Where's Alice's cell phone?  12. Where's the mouse?  13. Where is the student power?  14. Please help me find my glasses.  15. Please help me find my phone.  16. What is Bob doing now?  17. What is Alice doing now?  18. Improve the signals on the table.  19. Optimize the signals in the corridor.  20. Enhance the signals in the room.  21. Optimize the communication channel between Bob and Alice.  22. My cell phone cannot get a signal, please help me.  23. Enhance the Wi-Fi signal in the corridor.  24. Please check Alice's breathing.  25. Please check Alice's heartbeat.  26. Please check Alice's health status  27. What happened to Alice?  28. Localize the box 1 and 2.  29. Alice’s computer cannot get the signal, please help her.  30. Enhancing the communication link between router and robot A.  31. Monitor Alice's health condition.  32. Please check Bob's breathing and heartbeat.  33. Evaluate Bob's breathing and heartbeat.  34. Monitor Bob and Alice’s respiratory rate.  35. Examine Bob and Alice's respiration.  36. Check Bob's breaths.  37. Bob’s cell phone cannot get a signal.  38. Please check Bob's health status.  39. What is Bob doing now?  40. I want to know what Bob's doing now.  41. Please help me find the TV remote.  42. Please help me see what Alice is doing.  43. I can't find my glasses.  44. Get the position of yellow box.  45. Retrieve the present location of robot A.  46. Obtain the current coordinates of robot B.  47. I don't know where my glasses are.  48. My glasses seem to be missing.  49. I can't find my glasses.  50. Bob's computer cannot get the signals. |
| --- | --- |
| Complex instructions | 1. Please check the respiration rate of Bob and Alice.  2. Please check the heartbeat rate of Bob and Alice.  3. What are Bob and Alice doing?  4. Please send this picture to Alice’s computer.  5. Please send this picture to Alice and Bob’s computer.  6. Please check on Bob and Alice's health status.  7. Enhance the Wi-Fi signal in the corridor and room.  8. Localize all the boxes in the room.  9. Robot A, please go to the corridor.  10. Localize robot A and send the file to it.  11. Localize Bob, and send Bob’s position to robot A.  12. Let robot A move to yellow box.  13. Let robot B move to blue box.  14. Let robot A move to yellow box, and then move to blue box.  15. Let robot A move to yellow box, and pick up the wooden block.  16. Take Alice's key to the room.  17. Robot A, take me to the corridor.  18. Please send this picture to Alice's cell phone.  19. Let the robot A go to the pillbox and bring me my antihypertensive pills.  20. Alice needs help, please give her antihypertensive medication.  21. Alice requires assistance; please instruct the robot to fetch her antihypertensive medication.  22. Alice needs help; please have the robot deliver her blood pressure pills.  23. Please direct the robot to bring Alice her antihypertensive medication as she needs assistance.  24. Alice is in need of help; please instruct the robot to provide her with the antihypertensive medicine.  25. Assist Alice by having the robot deliver her the prescribed antihypertensive pills.  26. I need my glasses.  27. Robot A, please see me.  28. Robot A, come here.  29. Robot A, come to my location.  30. Robot A, move to this spot.  31. Robot A, come.  32. Robot A to the corridor.  33. Robot A, go to the table.  34. Let robot A and B go to room and corridor respectively.  35. Please give Alice the remote control I have here.  36. Come and help me take out the trash.  37. Robot A, follow me to the corridor.  38. Share the file with Bob first, then with Alice.  39. I'm thirsty. Please bring me the glass of water.  40. Forward pictures to Bob and Alice.  41. Share images with Bob and Alice.  42. Transmit photos to Bob and Alice.  43. Send Bob and Alice a picture.  44. Relay image to both Bob and Alice.  45. Let the robot A go to the pillbox and bring me my antihypertensive pills.  46. please bring Bob his antihypertensive pills.  47. Bob needs help, his pressure is really high.  48. Find my key and let the robot bring it to me.  49. If Bob and Alice are both in room A, send the file to them.  50. If Bob and Alice are both in room A, augment the communication link between them. |

**Supplementary Note 8. Skills of metaAgent**

Here, we detail the skills available in our proposed metaAgent system. As shown in **Table S9**, each of the 10 typical SPMs-based skills listed is based on the ability of the SPM to manipulate the electromagnetic manipulation of the environment. We have programmatically encapsulated each of these skills for coding expert to call upon. At the same time, metaAgent also supports user-defined skills, and we believe that more useful skills will be developed as metaAgent continues to be updated.

**Table S9 |** The Skills of metaAgent.

| Number | Skills |
| --- | --- |
| 1 | user_3d_skeleton_detection |
| 2 | user_localization |
| 3 | behavior_recognition |
| 4 | breath_detection |
| 5 | heartbeat_detection |
| 6 | communication_link_enhance |
| 7 | Information_transmission |
| 8 | object_localization |
| 9 | robot_localization |
| 10 | robot_control (move_robot_to\robot_arm_pickup\robot_arm_putdown\ robot_return) |

**(1)** **User 3D skeleton key-point detection:**

In order to detect human activities in real time, we use the 2.4 GHz metasurface system to monitor key-points of the human body. Our user key-point detection method adopts traditional supervised learning strategy, and its supervision data contain 3D coordinates of 34 human key-points generated by the ZED SDK. Each time the metasurface switches a coding pattern, the transmitting antenna emits a chirp signal with a bandwidth of 100 MHz and a duration of 5 μs, and the receiving antenna receives the scattered signal. In each measurement, 20 coding patterns are utilized to acquire microwave raw signals with the dimension of 2×20×64, where 20 is the number of the coding patterns, 64 represents the sampling points of received microwave signals per pattern, and 2 stands for the real/imaginary parts per sampling point. We then feed these microwave raw signals and corresponding supervised data into the SR-ANN ^[1]^ for learning, as shown in **Figure S6a**. The loss function is set to mean squared error (MSE). The batch size is set to 64, the learning rate is 0.001, and the number of epochs is set to 500. We collected over 50,000 data sets from 10 volunteers, involving various actions. After training, we deploy the pre-trained network on the computer to achieve real-time user key-point detection that meets human body monitoring requirements. **Figure S6b** shows the results of our experiments in the lab for the detection of key-points in the user's human skeleton.

**
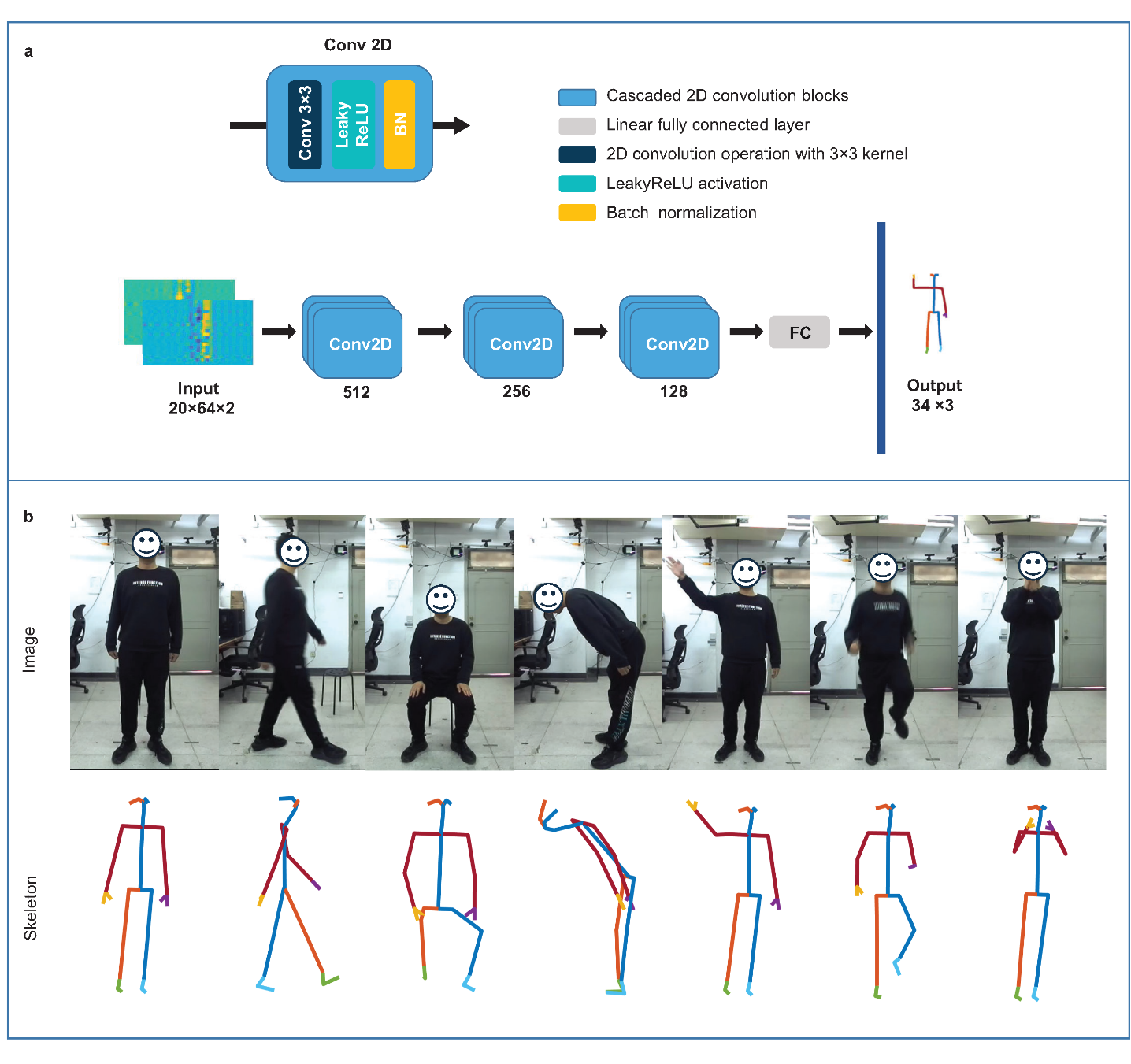
Figure S6** | (**a**)The SR-ANN’s architecture^[1]^. (**b**) Results of human 3D skeleton imaging.

**(2) User localization**

The user localization method directly uses the results of the user 3D skeleton key-point detection. The coordinates of the 34 key-points are averaged to obtain the user position. In order to mitigate the jitter and latency in positioning, we employ a one-euro filter to process the raw localization outcomes. The minimum cutoff frequency of the one-euro filter is set to 0.005, with a velocity coefficient of 0.8. We collected two localization results of users walking around in the laboratory, as shown in **Figure S7**.


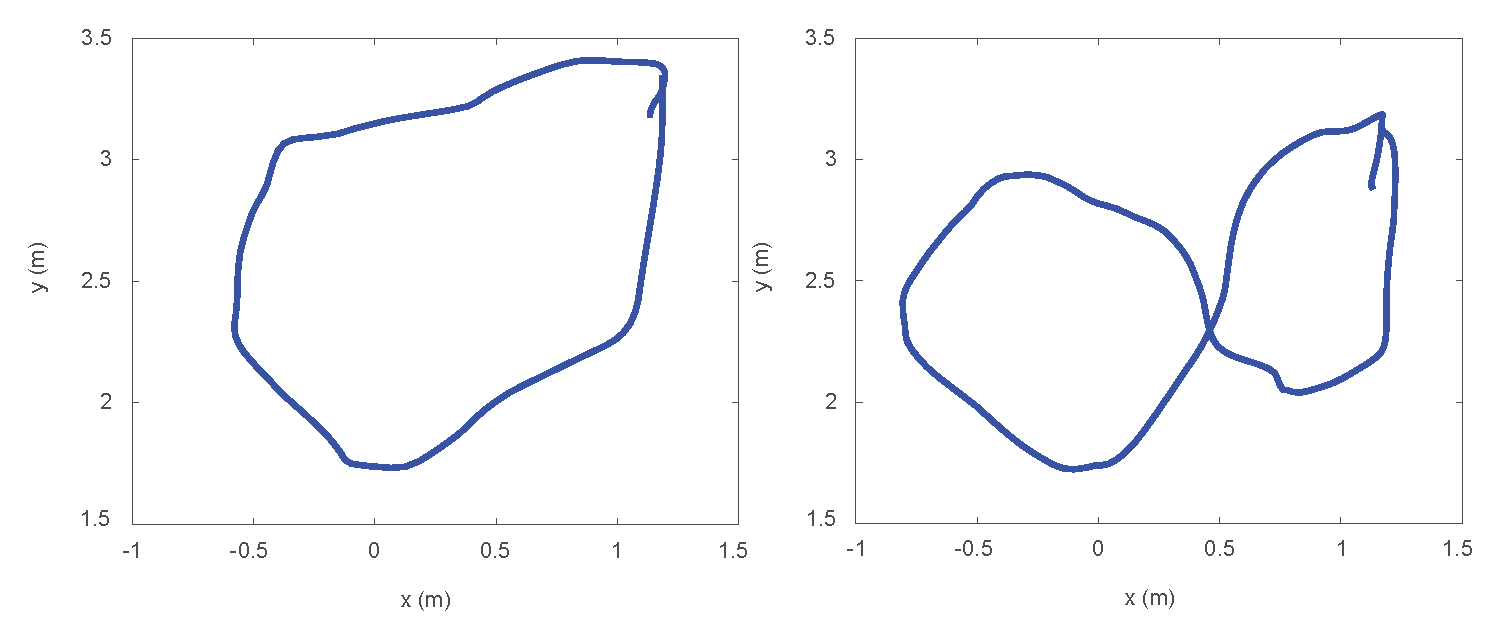


**Figure S7** | User localization results.

**(3) User behavior recognition**

The user behavior recognition skill recognizes the user's real-time behavior based on the user 3D skeleton key-point detection results and outputs it in semantic format. In order to obtain the semantic information of user behavior, we input the human body 3D key-point detection results into the BR-ANN^[1]^ algorithm so as to realize the classification of user behavior, as shown in **Figure S8a**. We designed 10 categories of common user behaviors indoors based on meta-agent application scenarios and collected the corresponding dataset for the training of this skill based on the microwave human 3D skeleton key-point detection results. The confusion matrix of the model is shown in **Figure S8b**. The experimental results are shown in **Figure S8c.**


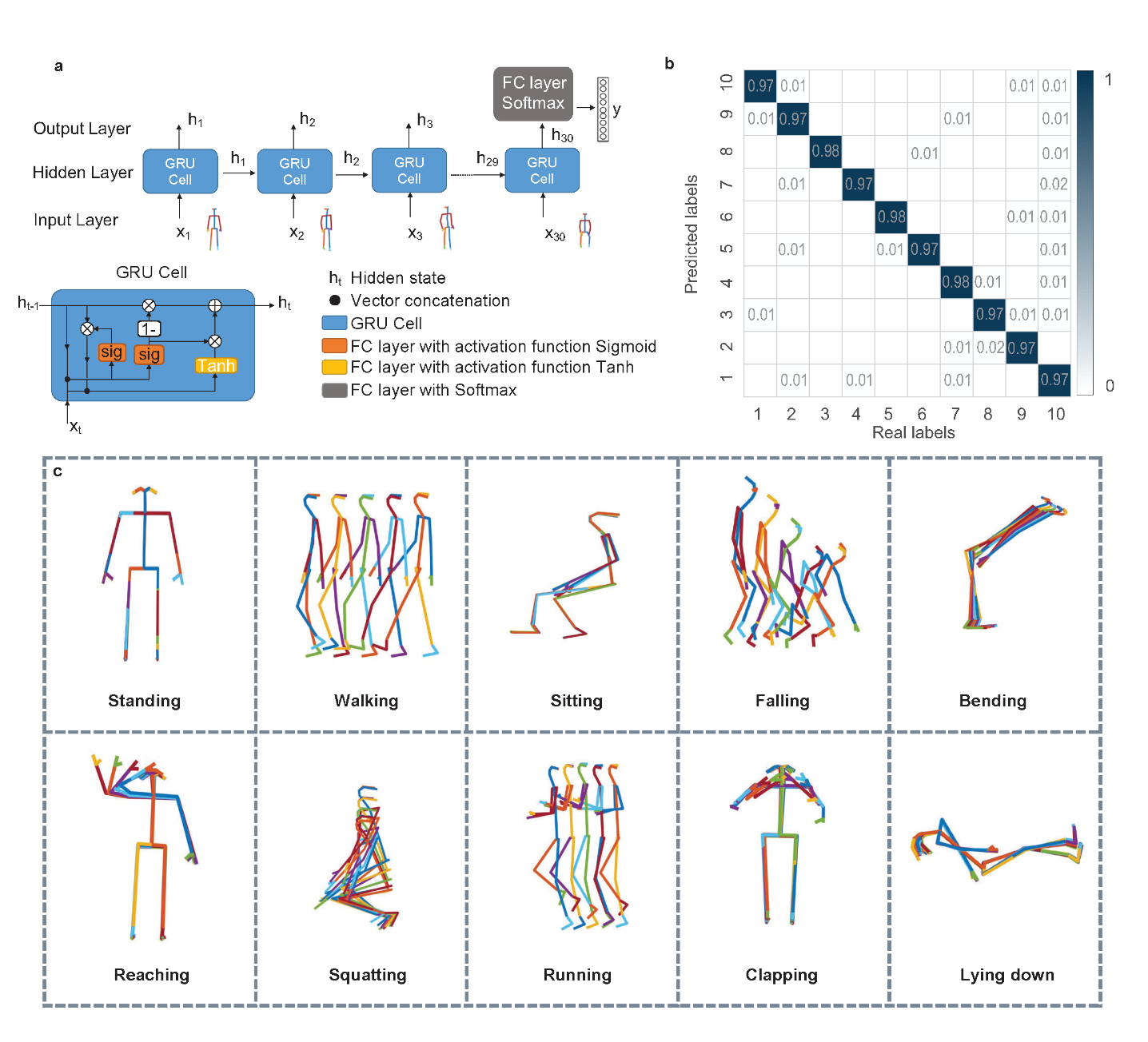


**Figure S8** | **(a)** The BR-ANN’s architecture^[1]^. (**b**) The confusion matrix**. (c)** Experimental results

**(4) Breath and heartbeat detection:**

Here we introduce metasurface-based detection of breath and heartbeat rate, as depicted in **Figure S9a**. Initially, microwave skeleton recognition is utilized to locate the position of the human chest cavity and corresponding focusing coding pattern is computed using the modified G-S algorithm. Subsequently, the transmitting antenna emits a Linear Frequency Modulation (LFM) signal with a bandwidth of 100 MHz every 0.037 s. Following the modulation of the metasurface, the beam can be precisely focused onto the human chest cavity, and the receiving antenna captures the scattered signals. The collected microwave signal is a time-frequency signal, from which we select a single-frequency signal. A window of 500 sampling points preceding the current moment is then selected for variational mode decomposition (VMD) to extract distinct components: DC signal, breath signal, and heartbeat signal. To mitigate spectrum leakage, the breath signal undergoes Hanning window processing, followed by Fast Fourier Transform (FFT) to obtain the breath spectrum. The predominant spectrum component within the range of 0.1 Hz-1 Hz is designated as the current breath rate, thereby enabling continuous monitoring of breath rates. Analogously, the heartbeat signal undergoes Hanning window application and FFT to derive the heartbeat spectrum, from which the maximum frequency component represents the current heart rate. **Figure S9b** shows the experimental results of breath and heartbeat detection across four distinct locations within the laboratory, demonstrating accurate detection of breath and heartbeat rate across varying positions.


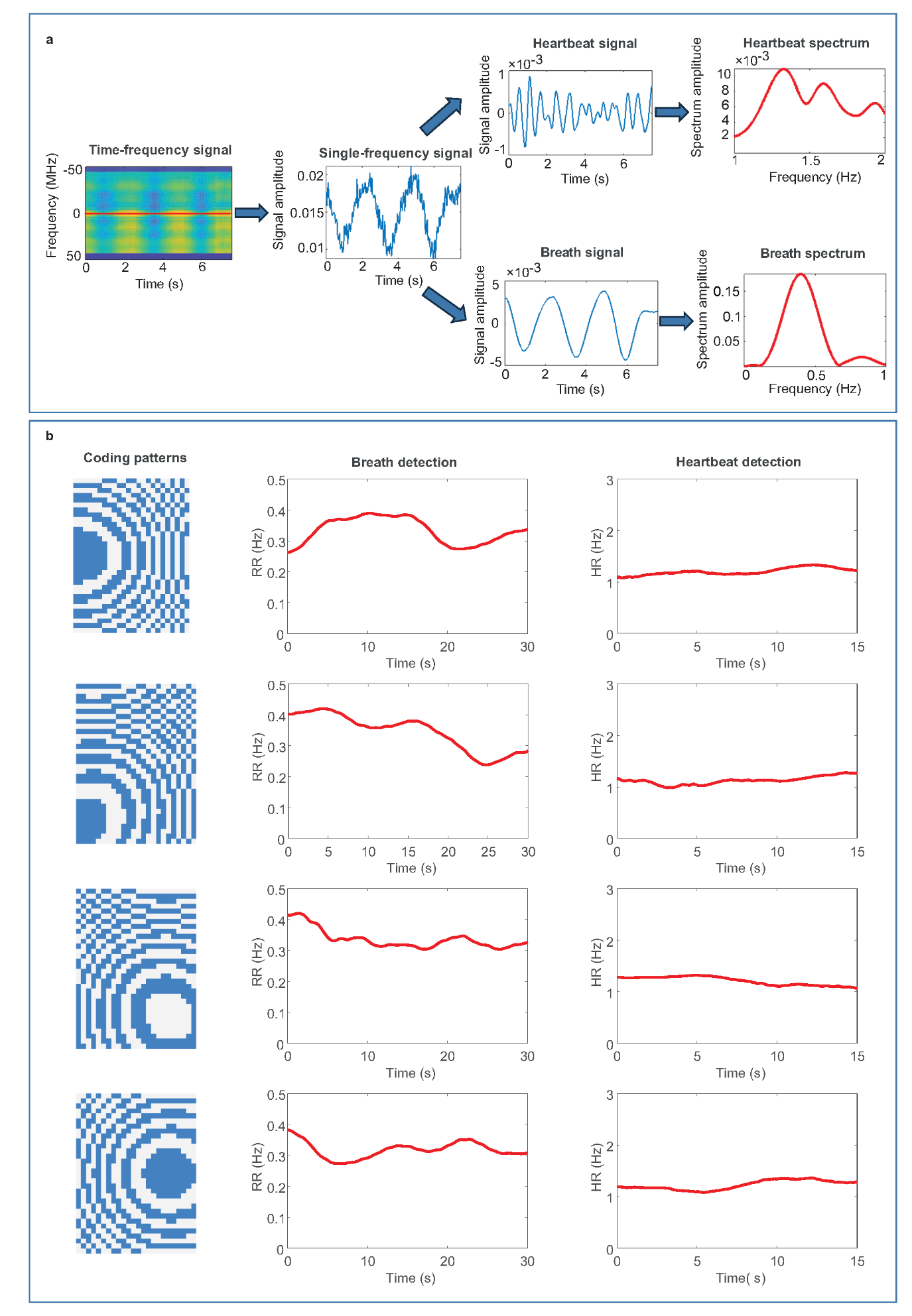


**Figure S9** | **(a)** Steps of metasurface-based detection of breath and heartbeat signals. (**b**)

Experimental results on breath detection and heartbeat detection across four distinct locations.

**(5) Communication link enhance and information transmission:**

In order to efficiently control the wireless devices in the system and achieve efficient information transmission, our metasurface-embodied agent uses the 2.4 GHz metasurface to support high quality wireless communication between devices. Relying on the capacity of metasurface to flexibly manipulate the electromagnetic energy distribution, the 2.4 GHz metasurface will enhance the wireless link at target devices designated by the agent, after that the transmitter will send the encoded signal to the receiver through the channel enhanced by metasurface. In addition, since the metasurface enhances the communication link directly from the physical layer, it can be easily embedded into existing communication systems. To test the performance of this metasurface-assisted communication system, we have experimented with BPSK and QPSK modulations, as we show in **Figure S10**. In **Figure S10a and b**, we respectively provide the real part of receiver’s BPSK and QPSK signal sequence with the metasurface enhancing the channel in the top half of the figure, and the corresponding constellation diagram in the bottom half of the figure. We also measure the RSSI of the receiver placed in different positions when the metasurface-assisted communication system adapts focus coding pattern to enhance the wireless link, and compare it to the situation with simply all-one coding metasurface. The experimental results are shown in **Figure S10c**. From the comparison result, we can see that the metasurface-assisted communication system can significantly improve the RSSI given the same transmitting power, thus guarantee that the metasurface-embodied agent is able to efficiently control wireless devices and perform high-quality information transmission.


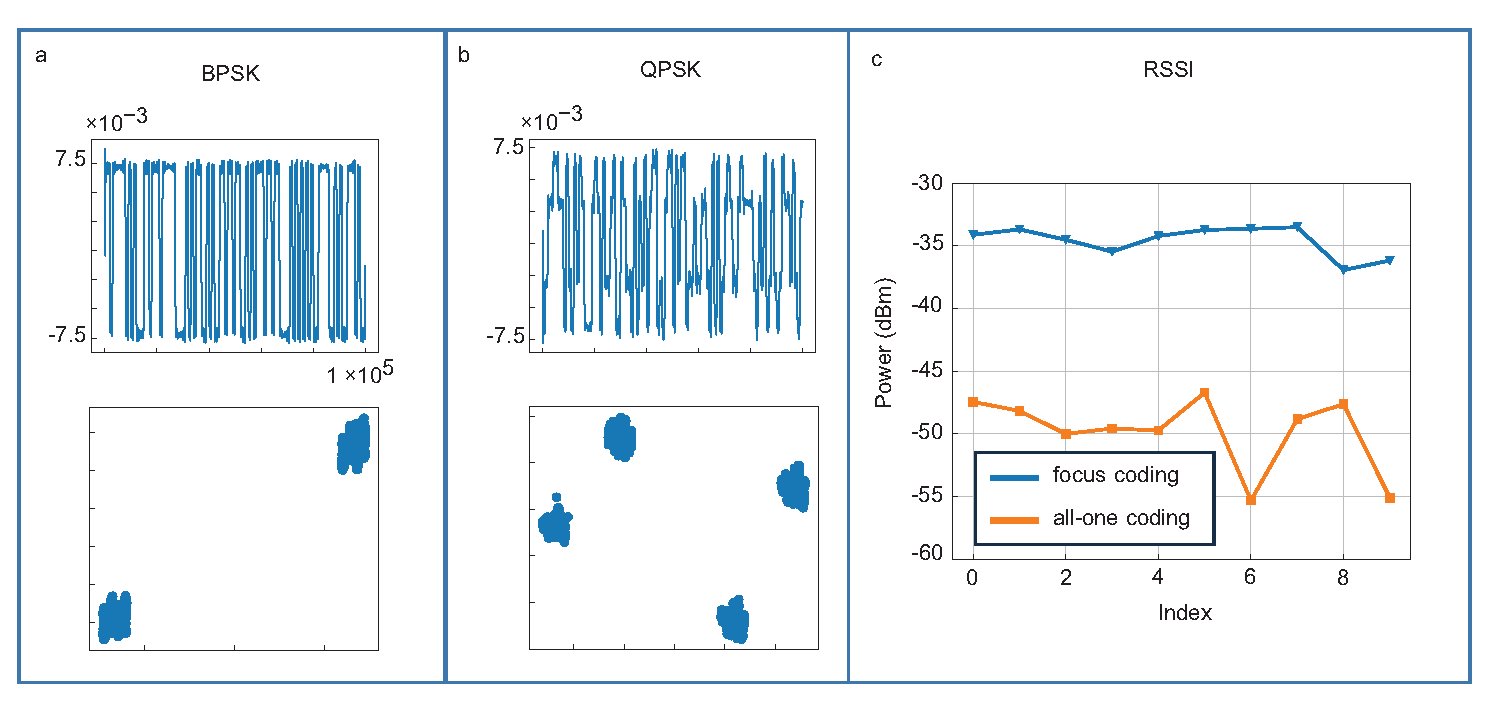


**Figure S10** | (**a**) the BPSK signal and constellation diagram of the receiver, (**b**) the QPSK signal and constellation diagram of the receiver, (**c**) the RSSI measurement result.

**(6) Object localization and robot localization:**

For the proposed metasurface-embodied agent, it’s a fundamental and practical capacity to localize the object and robot in the system conveniently. Therefore, we here adapt the hierarchical beam scanning method to implement near-field source localization for the cooperative and active object or robot based on the 2.4 GHz metasurface. The entire localization process can be summarized as follows: The region of interest (ROI) is divided into a number of 3D grids at a resolution of 0.1 m and we generate a two-stage codebook for the 2.4 GHz metasurface to execute the hierarchical beam scanning task. Before starting beam scanning, the transmitter sends a localization request to the target to be located according to the contents of the registration list, and establishes a connection with it. In beam scanning stage, as we show in **Figure S11a and b**, the metasurface will first switch its coding patterns to scan the whole ROI according to the pre-generated multibeam focused codebook, in which each coding pattern simultaneously selects three equally spaced 3D grids in the x-direction as targets for beam focusing, and measure the received signal strength indicator (RSSI) from the signal source at the same time. After that, a group of grids with high RSSI will be selected as subspace for refined scanning with the single-beam scanning codebook to focus the beam towards each grid in this subspace sequentially and measure the RSSI as well, then the grid points corresponding to the peaks of the RSSI values are judged as candidate solutions. After coordinate homogenization, the final localization result will be estimated from the candidate solutions.

In order to verify the effectiveness of the localization method, we randomly selected a number of target locations in a real indoor environment for the experiment, and the results are displayed in **Figure S11c**. An averaged three-dimensional localization error of about (0.07 m, 0.04 m, 0.16 m) is achieved with no complex functional requirements for other hardware other than basic RSSI measurement. To further demonstrate the results of localization, we show several sets of results in **Figure S11d-g**, where the left figure shows the coordinates of the 3D localization result and the actual position of the target, and the right figure shows the normalized received signal strength corresponding to the 2D grid slice closest to the localization result after filtering the candidate positions by the two-stage scanning judgement.


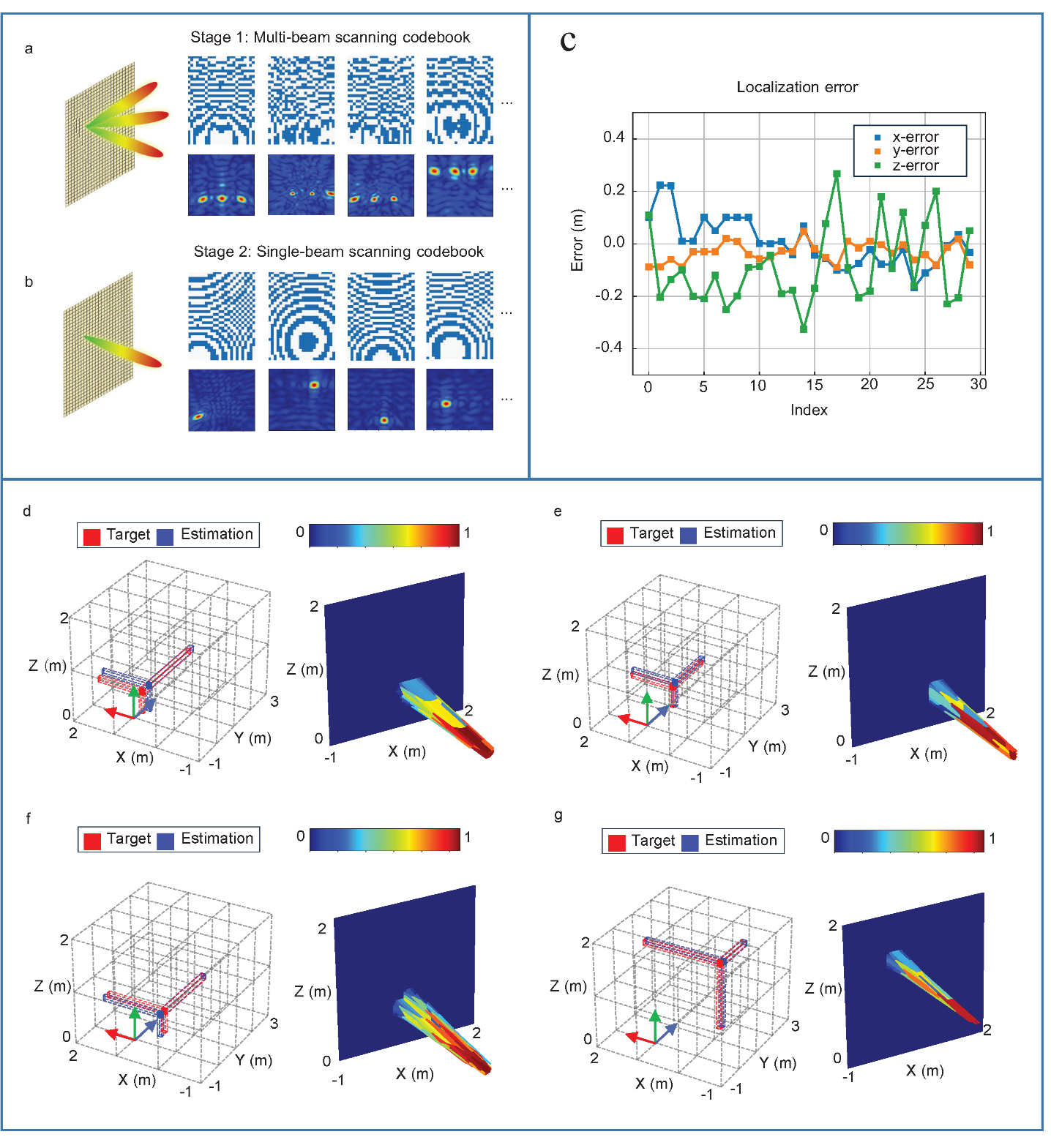


**Figure S11** | (**a**) multi-beam scanning stage, (**b**) single-beam scanning stage, (**c**) Experimentally measured localization errors, (**d-g**) Examples of the localization result.

**(7) Robot control:**

The robot used in this paper performs functions such as robot movement and robotic arm grasping based on the commands sent from the control center. The control center encodes the robot control commands and performs the communication enhancement introduced in **Supplementary Note 8 (5)** using the 2.4 GHz meatsurface to improve the quality of the Wi-Fi signal at the robot, and then sends the commands to the robot via a router to control the robot to perform the relevant functions. The command protocol for robot control is displayed in **Figure S12a**. In **Figure S12b-e**, we measured the sequence of real parts of the received signal corresponding to each control command and the constellation diagram after sampling processing, respectively.


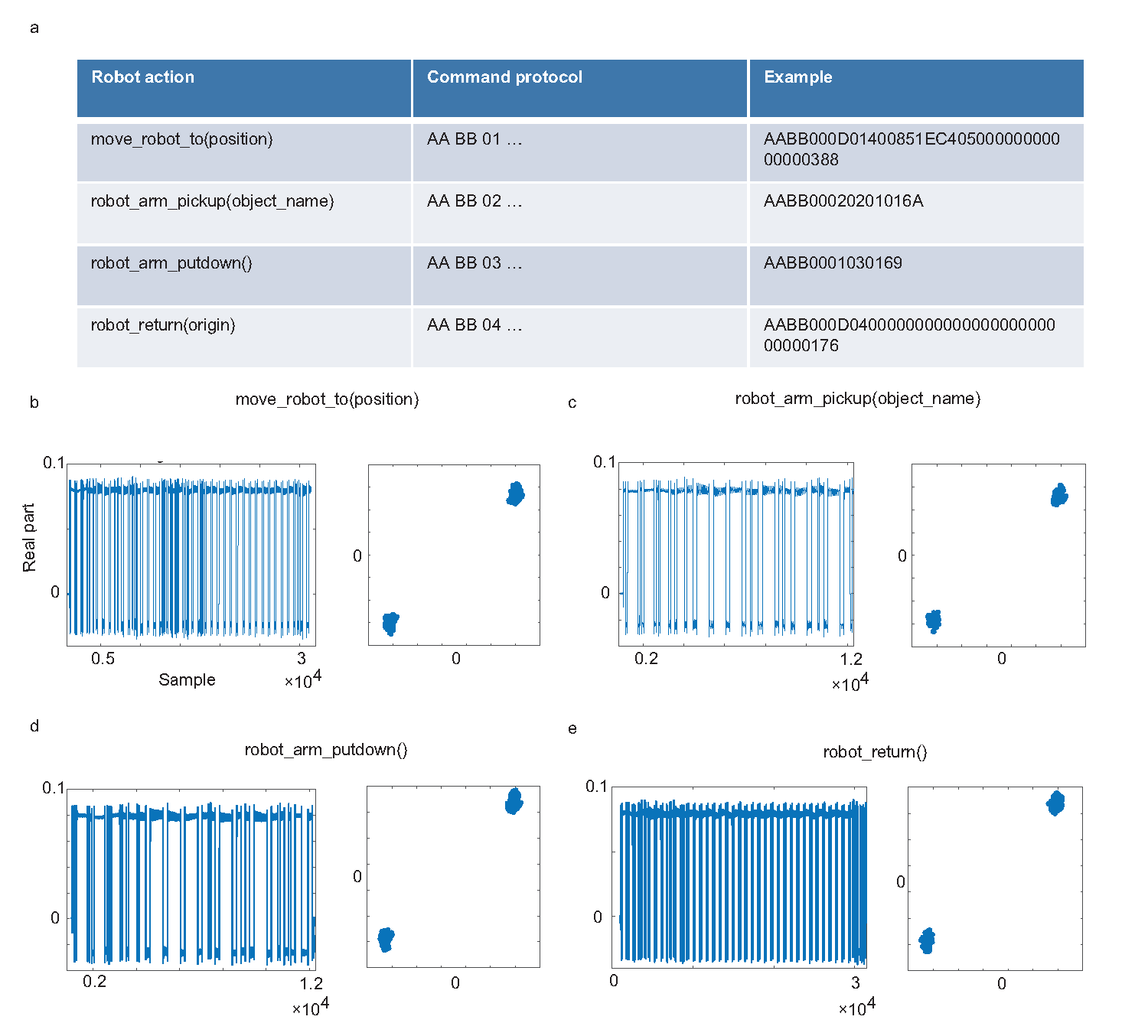


**Figure S12** | (**a**) robot control protocols, (**b-e**) real part and constellation diagram of the received signal corresponding to each control command

**Supplementary Note 9. Construction of knowledge graph (KG).**

Here, we introduce the construction process of knowledge graph in detail. At first, in osrder to obtain a priori information about the environment in which the metaAgent system operates, we reconstructed the indoor environment in 3D using a ZED2 camera and obtained the 3D environment map, as shown in in **Figure S13a**. Next, we performed semantic segmentation of the visual environment map based on different objects and labeled the corresponding semantics, thus generating a visual semantic map, as shown in **Figure S13b**. The VSM represents the position, orientation, and size of a static object in the environment, and we store the VSM in text format in the metaAgent's memory module for use by the metaAgent. Finally, in order to enable the metaAgent to better understand the environment and reason based on the knowledge of the environment, we performed entity extraction on the VSM, thus converting it into a knowledge graph format, as shown in **Figure S13c**. Converting it to KG format is more capable of providing the metaAgent with comprehensive details of each entity than the text format stored by the VSM, while also allowing entities to be associated with entities. This is highly convenient and accurate for metaAgent system to store and read knowledge.

**
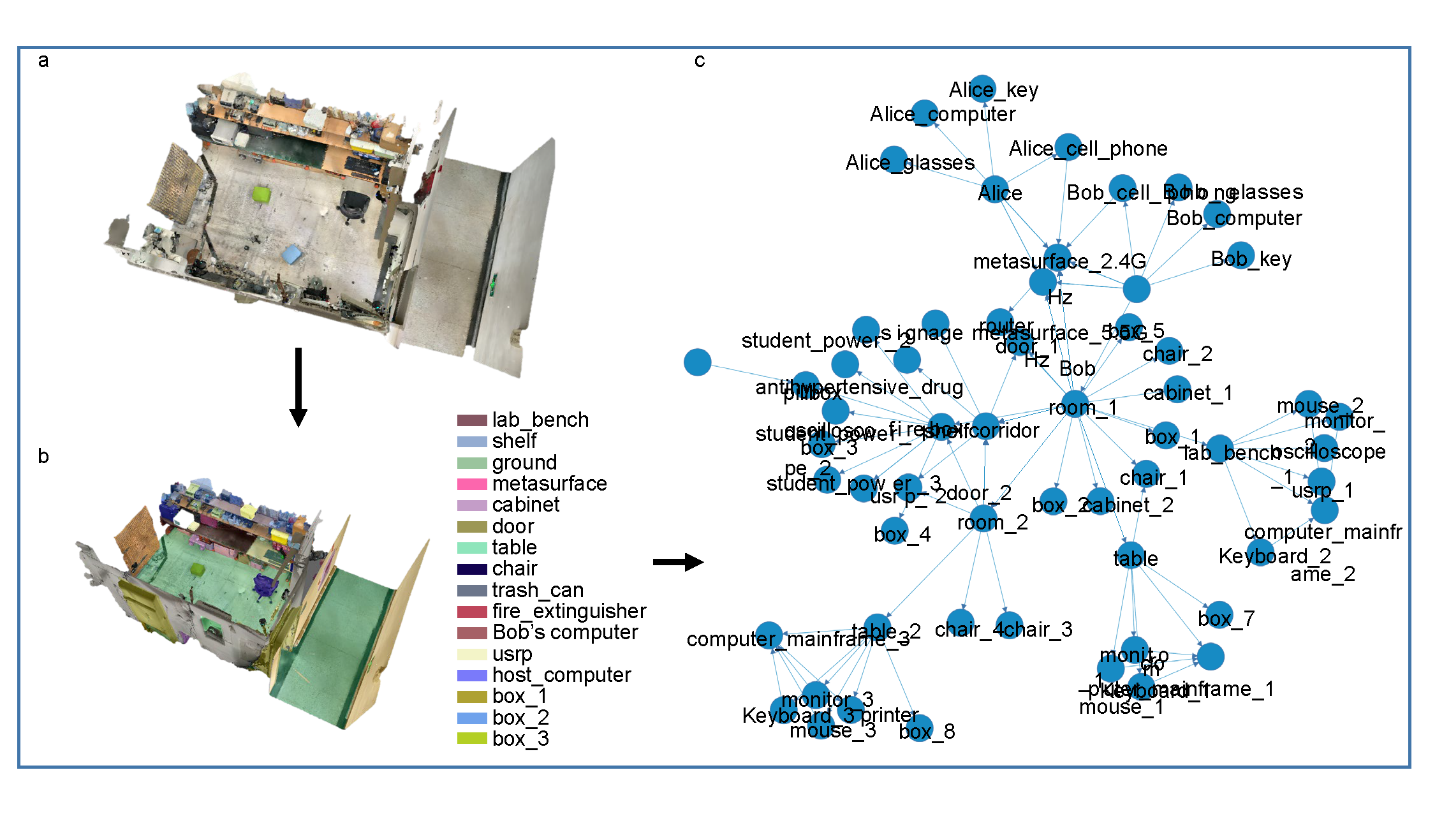
**

**Figure S13 |** (**a**) 3D environmental map, (**b**) visual semantic map (VSM), (**c**) knowledge graph (KG).

**Supplementary Note 10. Construction of memory module.**

Here, we describe the memory module in detail. Our proposed meta-agent system requires the use of two important knowledge bases in the cerebrum to accomplish the specification of high-level strategies, namely, the action and memory libraries. The action library is primarily used to store the executable actions of the MetaAgent, thus enabling the MetaAgent to select specific actions to accomplish for specific tasks when formulating advanced policies. With the action library, MetaAgent is capable of responding appropriately in different scenarios to perform a task or achieve a specific goal. The contents contained in action library are given in **Table S10a**, which mainly consists of a device library and a function library. For the device library, First, for the device library, it mainly contains all the devices that our MetaAgent can currently connect to manipulate. Here, it mainly includes two SPMs operating in the 2.4 GHz and 5.5 GHz bands, as well as a host computer. For the 2.4 GHz SPM, "SPM_2.4GHz", it integrates functions such as communication link enhancement, user localization, object localization, robot localization, and robot control. For the 5.5 GHz SPM, "SPM_5.5GHz", it integrates functions such as human breathing detection, user imaging, and information transmission. For the "host", it is mainly used to process some data storage and reading functions, such as searching for the position of the object in a semantic map. Here, the devices included in device library can be easily added according to the actual application scenarios. For the function library, it contains a detailed description of how to use each function in detail, as well as the inputs and outputs required to provide the MetaAgent with the ability to perform task decomposition and make reasonable actions. In addition, some necessary tips on usage precautions will also provide the necessary complementary information for MetaAgent's strategy formulation. The memory library is used to store the MetaAgent's past experiences, learnings and observations. It allows the MetaAgent to learn from past experiences and adapt to new situations. In addition, the information stored in the memory library about the physical environment, etc., allows the MetaAgent to better understand and deal with similar future situations, thus enabling the MetaAgent to make strategies that reach the level of human intelligence based on past experiences during the process of performing autonomous strategy formulation. Here, the memory library that we provide for the MetaAgent contains mainly a priori visual semantic maps built in advance in the environment in which it is applied and a set of basic common-sense knowledge of human feedback. As shown in **Table S10b**, we provide MetaAgent with the memory library which is mainly divided into environmental memory and common-sense memory. Among them, the environment memory contains a priori visual semantic maps established in advance in the environment applied by MetaAgent; the common-sense memory contains a series of work-based common-sense knowledge and useful human feedback experience knowledge.

**Table S10** | The memory module utilized by our proposed MataAgent. **(a)** action library; **(b)** memory library

| **(a) {Action Library}**  """  MetaAgent can modulate electromagnetic waves in indoor space to accomplish tasks such as communication link enhance, human localization, breath detection, send file, object localization, signal enhancement, and robotic control.  ## {Device Library}  The available devices and the functions they have are shown below:  """  <<device library>>  """  s  ## {Function Library}  This library lists the basic function you can use to solve task.  """  **<<**function library**>>**  """  A few useful things:  (1) The field "device_name" must be filled with the device having this function according to {Device Libray}.  (2) Only one function can be selected per subtask step.  (3) Before use function "communication_link_enhance", two steps are needed to determine the location of the source and the location of the target, respectively. First, use function "search_object_from_KG_VSM" to get the location of the 'router' from the semantic map, and get the location of the target in parallel.  {User Library}  The users which have registered with id in the system are listed as follows: ['Alice', 'Bob']  {Robot Library}  The robots which have registered with id in the system are listed as follows: ['Robot_A':'11','Robot_B':'22']  """ |
| --- |
| **(b) {Memory Library}**  """  The memory library is organized into two parts: {common-sense Library} for storing some additional common-sense information from user feedback for metaAgnet; {Environment Library} for storing information about objects, users, and robots present in the environment.  ##2.1 {common-sense Library}  When you have completed your reasoning task you need to check that the result satisfies the following basic common sense:  The following common-sense memories are provided for you to use when reasoning:  <<common_sense>>  ##2.2 {Environment Library}  The following objects are in the visual semantic map (VSM) and knowledge graph (KG), and you should refer to them using these exact names:  """  << KG-VSM >>  """  A few useful things:  (1) The above objects represent that all exist and their positions are known.  (2) When you perform task inference, you need to first determine if the object already exists in the above semantic map.If the object exists in the semantic map, the "search_object_from_KG_VSM()" function is used to obtain its location; \  if it does not exist, you need to use the "object_localization()" function to search for the location of the unknown object.  (3) The following objects are uncertain in position, and you are to refer to them using these exact names:  """  'Bob_cell_phone','Alice_cell_phone','Bob_key','Alice_key','TV_remote','Bob_glasses','Alice_glasses'  """ |
| **<<device libray>>**  [  {  "device_name": "SPM_2.4GHz",  "functions": [  "communication_link_enhance",  "user_3d_skeleton_detection",  "user_localization",  "object_localization",  "robot_localization",  "move_robot_to",  "robot_arm_pickup",  "robot_arm_putdown",  "robot_return",  "information_transmission"  ]  },  {  "device_name": "SPM_5.5GHz",  "functions": [  "breath_detection",  "heartbeat_detection",  "behavior_recognition"  ]  },  {  "device_name": "host",  "functions": [  "search_object_from_KG_VSM",  "voice_interaction",  "emergency_call"  ]  }  ] |
| **<<function library>>**  (1) meta.search_object_from_KG_VSM(object_name) - Search the object on the {Environment Library}, input the string of object_name and return the position (x, y, z).  (2) meta.user_3d_skeleton_detection(user_position) - Recognize the behavior of the user in the inputed uesr position, return the user's 3D skeleton sequence (Numpy array of size 20*34*3).  (3) meta.user_localization(user_name) - Localize the user registering in {User Library},and return a list indicating the position x, y, z.  (4) meta.behavior_recognition(user_position) - Recognize user behavior based on user's position , return the current behavior of the user as a string.  (5) meta.breath_detection(user_position) - Detect the breath rate of user in the input user’s position , and return a float data indicating the breath rate.  (6) meta.heartbeat_detection(user_position) - Detect the heartbeat rate of user in the input user’s position , and return a float data indicating the heartbeat rate.  (7) meta.communication_link_enhance(source, target) - Enhance the quality of wireless communication to the target location, the inputs are source location and target location, return True if successful.  (8) meta.object_localization(object_name) - Localize the object which is not exist in {semantic_map}, and return the position (x, y, z).  (9) meta.robot_localization(robot_name, id) - Localize the robot registering in {Robot Library},and return the position (x, y, yaw).  (10) meta.move_robot_to(position) - Move robot to the specified position, return nothing.  (11) meta.robot_arm_pickup(object_name) - Pick up the specified object, return nothing.  (12) meta.robot_arm_putdown() - Put down the specified object, return nothing.  (13) meta.robot_return() - Robot returns to original position, return nothing.  (14) meta.information_transmission(object_name) - Send the contents of a specified folder to a specified object.  (15) meta.voice_interaction(contents) - Returns the user's reply (string) after voice playing the contents(string) out and activating the microphone to wait for the user's reply.  (16) meta.emergency_call() - Call for emergency medical assistance.  (17) … |
| **<<common_sense>>**  (1) Subtasks that require different devices can be executed in parallel, while subtasks that require the same device can only be executed sequentially  (2) If the two subtasks have no dependencies on each other and use different devices, they can be executed in parallel.  (3) For "communication_link_enhance" function the source and target locations must be determined first, and therefore need to be decomposed into three subtasks in order to be accomplished  (4) Before you use the "move_robot_to" and "robot_arm_pickup" functions,you first need use "communication_link_enhance" function to enhance the signal for Robot A from router.  (5) Before use function "communication_link_enhance", two steps are needed to determine the location of the source and the location of the target, respectively. First, use function "search_object_from_KG_VSM" to get the location of the 'router' from the environment library, and get the location of the target in parallel.  (6) Getting the 'router' position also requires a separate subtask to accomplish.  (7) A single device cannot execute multiple functions at the same time.  (8) To detect the user's breathing it is only necessary to locate the user's position.  (9) When user's pressure rises, you need to control robot to bring him/her antihypertensive pills right away.  (10) If the antihypertensive pills been delivered successfully, you can end this task and Reply 'END'.  (11) If the task is complete, then you should voice ask the user again if they need additional help. If the user replies that he or she does not need help, then end this task and Reply 'END'.  (12) Bob is used to calling the robot to his position with "See me."  … |
| **<< KG-VSM >>**  {  'origin','chair_1','room', 'corridor', 'lab_bench', 'table', 'shelf', 'chair', 'yellow_box', 'green_box', 'blue_box', 'cabinet_1', 'cabinet_2',  'metasurface_2.4G', 'metasurface_5.5G', 'metasurface_9.7G', 'meter_box', 'door_1', 'door_2', 'box_1', 'box_2', 'box_3', 'box_4',  'box_5', 'box_6', 'box_7', 'pillbox', 't_antenna', 'r_antenna', 'trash_can', 'oscilloscope', 'usrp', 'host_computer_1', 'host_computer_2',  'student_power_1', 'student_power_2', 'student_power_3', 'Alice_computer', 'Bob_computer', 'router', 'fire_extinguisher', 'wlooden_block', 'antihypertensive_drug', ….  } |

**Supplementary Note 11. Experiments on metaAgent support for Chinese language commands.**

Here, we perform an experimental evaluation of the metaAgent system's ability to support other languages. In this experiment, we choose to use Chinese language as the test language, mainly testing metaAgent about the success rate of task execution with Chinese instructions as input. We selected 10 simple and 10 complex commands for experimental validation, and each command was run 20 times individually. The LLM used is the “GPT-3.5 turbo” model. The experimental results are shown in **Table S11**. The results show that metaAgent can support the correct understanding of Chinese commands, and all the success rate indicators can reach the level of English commands.

**Table S11** | Success rates of our metaAgent over 20 Chinese language commands with different complexities.

| **Instructions (Chinese)** | | **Steps** | **Success Rate (%)** | | | |
| --- | --- | --- | --- | --- | --- | --- |
|  |  |  | **TP** | **SG** | **CG** | **TE** |
| **Simple instructions** | 桌子的位置在哪？ | 1 | 100 | 100 | 100 | 100 |
|  | 爱丽丝(Alice)在哪？ | 1 | 100 | 100 | 100 | 90 |
|  | 我的手机在哪？ | 1 | 100 | 100 | 85 | 80 |
|  | 机器人 A 在哪里？ | 1 | 95 | 100 | 95 | 90 |
|  | 请找到路由器的位置。 | 1 | 100 | 95 | 90 | 80 |
|  | 请检查鲍勃（Bob）的呼吸。 | 2 | 95 | 100 | 95 | 85 |
|  | 爱丽丝(Alice)怎么了？ | 2 | 85 | 100 | 95 | 80 |
|  | 请检查鲍勃(Bob)的健康状况。 | 2 | 95 | 95 | 90 | 80 |
|  | 增强走廊的 Wi-Fi 信号。 | 3 | 100 | 100 | 95 | 90 |
|  | 我的手机收不到信号，请帮帮我。 | 3 | 95 | 100 | 95 | 85 |
|  | **Overall (10)** |  | **96.5** | **99.0** | **94.0** | **86.0** |
| **Complex instructions** | 请检查鲍勃(Bob)和爱丽丝(Alice)的呼吸频率。 | 4 | 100 | 100 | 95 | 90 |
|  | 鲍勃(Bob)和爱丽丝(Alice)在做什么？ | 4 | 95 | 100 | 95 | 80 |
|  | 机器人 A，请到我这里来。 | 5 | 90 | 95 | 95 | 75 |
|  | 机器人 A，带我去走廊。 | 7 | 95 | 100 | 95 | 70 |
|  | 请将这张图片发送到爱丽丝(Alice)的电脑上。 | 4 | 85 | 100 | 85 | 85 |
|  | 让机器人A去药箱，把我的降压药拿来。 | 8 | 80 | 95 | 75 | 65 |
|  | 请把鲍勃的降压药拿来。 | 8 | 80 | 95 | 80 | 65 |
|  | 让机器人 A 把鲍勃（Bob）手中的木块拿给我。 | 9 | 75 | 95 | 75 | 75 |
|  | 让机器人 A 和机器人B 分别去房间和走廊。 | 10 | 80 | 100 | 90 | 75 |
|  | 鲍勃(Bob)需要帮助，他现在的血压很高。 | 9 | 80 | 90 | 80 | 65 |
|  | **Overall (10)** |  | **86.0** | **97.0** | **86.5** | **74.5** |

**References**

[1] Wang, Zhuo, et al. Multi‐Task and Multi‐Scale Intelligent Electromagnetic Sensing with Distributed Multi‐Frequency Reprogrammable Metasurfaces. *Advanced Optical Materials* **12**.6: 2203153(2024).
